# Mechanism of DNA cleavage by the endonuclease SauUSI: a major barrier to horizontal gene transfer and antibiotic resistance in *Staphylococcus aureus*

**DOI:** 10.1093/nar/gkab042

**Published:** 2021-02-03

**Authors:** Vinayak Sadasivam Tumuluri, Vrunda Rajgor, Shuang-Yong Xu, Om Prakash Chouhan, Kayarat Saikrishnan

**Affiliations:** Department of Biology, Indian Institute of Science Education and Research, Pune 411008, India; Department of Biology, Indian Institute of Science Education and Research, Pune 411008, India; New England Biolabs Inc., Research Department, Ipswich, MA 01938, USA; Department of Biology, Indian Institute of Science Education and Research, Pune 411008, India; Department of Biology, Indian Institute of Science Education and Research, Pune 411008, India

## Abstract

Acquisition of foreign DNA by *Staphylococcus aureus*, including vancomycin resistance genes, is thwarted by the ATP-dependent endonuclease SauUSI. Deciphering the mechanism of action of SauUSI could unravel the reason how it singularly plays a major role in preventing horizontal gene transfer (HGT) in *S. aureus*. Here, we report a detailed biochemical and structural characterization of SauUSI, which reveals that in the presence of ATP, the enzyme can cleave DNA having a single or multiple target site/s. Remarkably, in the case of multiple target sites, the entire region of DNA flanked by two target sites is shred into smaller fragments by SauUSI. Crystal structure of SauUSI reveals a stable dimer held together by the nuclease domains, which are spatially arranged to hydrolyze the phosphodiester bonds of both strands of the duplex. Thus, the architecture of the dimeric SauUSI facilitates cleavage of either single-site or multi-site DNA. The structure also provides insights into the molecular basis of target recognition by SauUSI. We show that target recognition activates ATP hydrolysis by the helicase-like ATPase domain, which powers active directional movement (translocation) of SauUSI along the DNA. We propose that a pile-up of multiple translocating SauUSI molecules against a stationary SauUSI bound to a target site catalyzes random double-stranded breaks causing shredding of the DNA between two target sites. The extensive and irreparable damage of the foreign DNA by shredding makes SauUSI a potent barrier against HGT.

## INTRODUCTION

Antimicrobial resistance (AMR) is an outstanding threat to the health care system worldwide (1). One serious concern is the resistance in *Staphylococcus aureus—*an opportunistic pathogen responsible for the majority of the nosocomial and community-acquired infections in humans. Presently, the first-line of treatment involves antibiotics penicillin and methicillin. However, over the years, many strains of *S. aureus* have grown resistant to them (2–4). Vancomycin is a glycopeptide antibiotic that is known to treat methicillin-resistant *S. aureus* (MRSA). The ability of MRSA to acquire vancomycin-resistance through horizontal gene transfer (HGT) is a looming health crisis (5,6). HGT is the process of acquisition of foreign DNA by bacteria through transduction, transformation or conjugation, and is a biological driver of bacterial evolution, ecology and pathogenicity (7,8). Restriction endonucleases (REases) that nucleolytically degrade foreign DNA are one of the main barriers to HGT in bacteria (9,10). A striking example is an almost impregnable barrier created by the REase SauUSI to the entry of foreign DNA into *S. aureus* (11–13).

SauUSI was first discovered in the MRSA strains UAMS-1 and SA564, and was found to be one of the two main barriers to HGT in many strains of *S. aureus*; the REase Sau1 being the other (11,12,14,15). Based on the epigenetic status of their substrate DNA, REases are broadly classified as modification-independent Type I, ISP, II or III REases or modification-dependent Type IV REases (16). Modification-independent REases cut DNA having non-modified target sites, while modification-dependent REases cleave DNA only if the target sites have a specific modified base. SauUSI is a modification-dependent Type IV REase, and Sau1 is a modification-independent Type I REase. The dynamics of the REases, i.e. the expression levels of REases, the types of the REases acting in tandem and their nucleolytic potential in the bacterial cell, dictate the rate of HGT and thus potentially the ability of a bacterium to gain resistance to an antibiotic. Inactivation of the *sauUSI* gene leads to a manifold increase in the transformation efficiency of *S. aureus* (11,17,18). Interestingly, deletion of *sau1* in these strains does not have such a profound effect (11,13,19). Furthermore, the absence of SauUSI results in hyper-susceptibility to the gain of the vancomycin resistance genes from *Enterococcus faecalis* (11,13). What makes SauUSI such a potent barrier to HGT is not clear.

SauUSI is made of a single polypeptide chain containing three domains - a nuclease domain belonging to the phospholipase D (PLD) family, an ATPase domain belonging to the Superfamily 2 (SF2) of helicase, and a target recognition domain (TRD) (11,12). The Type IV REase SauUSI cleaves DNA only in the presence of the target sequence 5′-S^5m^CNGS-3′, where S = G or C and ^5m^C can be 5-methylcytosine or 5-hydroxymethylcytosine (12). It was previously reported that SauUSI cleaves only those DNA having two or more target sites. Based on the site of their cleavage, REases can be categorized as site-specific or random cutters. Site-specific REases, such as Type II and Type III REases, cleave inside or at a fixed distance outside the target sequence. In contrast, the random cutters, such as Type I and ISP REases, cut randomly as far away as thousands of base pairs (bp) from their target sequence. Preliminary analysis showed that SauUSI cleaves DNA 3–18 bp outside of the target sequence (12).

SauUSI requires ATP as a cofactor for its nucleolytic activity (12). REases that require ATP as a cofactor for DNA cleavage usually belong to Type I, ISP or III (20,21). In the case of Type I and ISP REases, ATP hydrolysis powers translocation of the enzyme along the DNA that allows it to cleave DNA away from the target sequence, while the hydrolysis of ATP by Type III REases primarily serves as a switch to turn on its nucleolytic activity (20,22–24). The role of ATP hydrolysis in DNA cleavage by SauUSI is unknown. Here, through complementary biochemical and structural studies, we unravel the mechanism of DNA cleavage by SauUSI and the role of ATP hydrolysis for its nucleolytic activity and gain insights into what makes this enzyme a potent barrier to HGT.

## MATERIALS AND METHODS

### Cloning and expression of SauUSI

The *sauUSIR* gene followed by a BamHI site and C-terminal 6xHis-Tag downstream and flanked by a NdeI site upstream was inserted into a pHis17 (ampicillin resistant) vector by a PCR based restriction-free cloning strategy. NEB turbo electrocompetent cells were transformed with the plasmid DNA. The positive clone was confirmed by sequencing for the presence of the wild type *sauUSIR* gene. Small scale expression of SauUSI suggested that it was best expressed in BL21(AI) cells grown in LB (lysogeny broth) supplemented with ampicillin (0.1 mg/ml) up to 0.6 OD at 37°C, induced with 0.2% arabinose and left to grow overnight at 16°C.

### Purification of SauUSI for biochemical assays

The purification of SauUSI was performed using a three-column strategy at 4°C. Two liters culture was pelleted down using an Avanti J26X-SP at an RCF of 5180 for 20 min. The pellet was resuspended in a lysis buffer (50 mM Tris–HCl, pH 8, 500 mM NaCl, 5 mM MgCl_2_, 25 mM imidazole, 10% glycerol and 0.2% CHAPS) and lysed in a sonicator for 5 min using a pulse of 1 s and recovery of 3 s at a 60% amplitude. Clarification of the cell lysate was achieved by ultra-centrifuging the sample at 100 000 g using an Optima-L-100K ultracentrifuge. The supernatant was loaded onto a GE HisTrap™ HP 5 ml column and washed thoroughly with Buffer A (50 mM Tris–HCl, pH 8, 500 mM NaCl and 25 mM imidazole). Elution of the protein was achieved by using a step gradient of Buffer B (50 mM Tris–HCl, pH 8, 500 mM NaCl and 500 mM imidazole). Presence of protein was confirmed using a 10% SDS-PAGE gel, and the appropriate fractions were pooled for further processing. The pooled fractions were dialyzed against Buffer B0 (50 mM Tris–HCl, pH 8, 1 mM EDTA and 1 mM DTT) using Snakeskin dialysis tubing 10 000 MWCO for 3 h. The dialyzed sample was then loaded onto a GE MonoQ™ 10/100 GL column using Buffer B50 (50 mM Tris–HCl, pH 8, 50 mM NaCl, 1 mM EDTA and 1 mM DTT). Elution of the protein was achieved using a linear gradient (0%-50%) of Buffer B1000 (50 mM Tris–HCl, pH 8, 1000 mM NaCl, 1 mM EDTA and 1 mM DTT). The fractions containing protein (confirmed by a 10% SDS-PAGE gel) were pooled and concentrated using a Vivaspin Turbo 10K MWCO centricon. The concentrate was loaded onto a GE Superdex 200™ increase 10/300 GL column, and the protein was eluted using Buffer B100 (50 mM Tris–HCl, pH 8, 100 mM NaCl and 1 mM DTT). Pure protein was analyzed on a 10% SDS-PAGE gel, concentrated appropriately and plunge frozen using liquid nitrogen and stored at –80°C for biochemical studies (Supplementary Figure S1).

### DNA substrates used for cleavage assay

**Figure 1.**
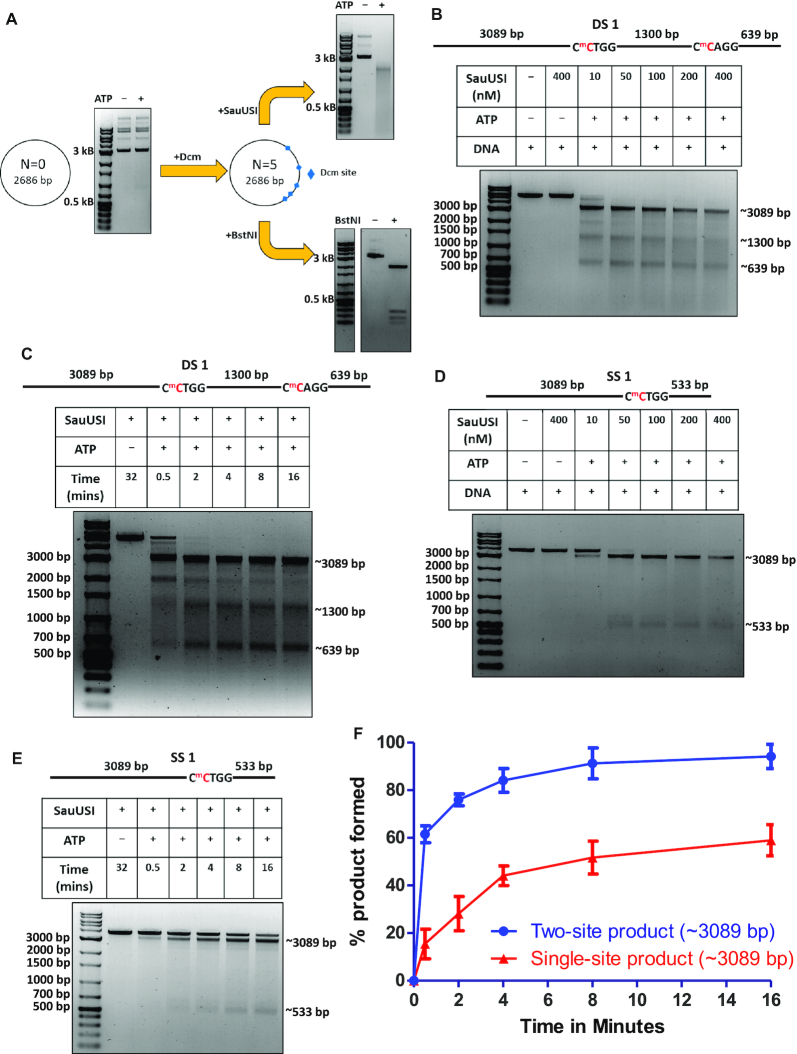
Nuclease activity of SauUSI. (**A**) Schematic of the plasmid used for cleavage. N represents the number of target sites of SauUSI in a particular plasmid. Methylation was brought about *in vivo* to generate the target sites of SauUSI. The corresponding cleavage pattern (on a 1% agarose gel) of SauUSI on the respective plasmids. The reactions with and without nucleotide (ATP) are compared against a DNA marker. (**B**) The two-site substrate (DS 1) used for the cleavage assay; the separation between the two sites is 1300 bp. The cytosine highlighted in red represents methylation. The target site is methylated on both the strands. Representative 1% agarose gel for two-site cleavage using varying concentrations (10–400 nM) of SauUSI. (**C**) Single-site substrate (SS 1) used for the cleavage assay. The cytosine highlighted in red represents methylation. Methylation is present on both the strands. The target site is methylated on both the strands. Representative 1% agarose gel for single-site cleavage using varying concentrations (10–400 nM) of SauUSI. (**D**) Representative 1% agarose gel for two-site cleavage (DS 1) carried over a time period of 30 s to 16 min. (**E**) Representative 1% agarose gel for single-site (SS 1) cleavage carried over a time period of 30 seconds to 16 min. (**F**) Percentage of product (the ∼3089 bp band) formed as a function of time (DNA concentration 3 nM and protein concentration 100 nM) (*n* = 3). In the case of DS 1, the ∼3089 bp fragment can arise from a single-site cleavage (at the left hand side) or from a sequential two-site cleavage.

The substrates used in the cleavage assays were plasmids and methylation was achieved *in vivo* using *dcm^+^* DH5α *Escherichia coli* and *in vitro* using commercial M.MspI (NEB). Dcm recognizes the DNA sequence 5′-CCAGG-3′ and converts it to 5′-C^5m^CAGG-3′ whereas M.MspI recognizes the DNA sequence 5′-CCGG-3′ and converts it to 5′-^5m^CCGG-3′. The sequences methylated by Dcm or M.MspI are subsets of the SauUSI target site 5′-S^5m^CNGS-3′. Methylation and non-methylation were achieved by transforming plasmids into DH5α *E. coli* and NEB Turbo respectively. Plates were kept overnight at 37°C, and single colonies were picked and inoculated in LB (0.1 mg/ml of ampicillin) and grown until turbidity. The cells were pelleted down, and plasmid preparation was performed using QIAprep® miniprep spin protocol. The plasmid represented in Figure 1A is a derivative of pUC18. The two-site substrate 1 (DS 1) and the three-site substrate (TS) are derivatives of the pHis17 vector which were linearized with NcoI-HF at 37°C for 1 h according to the NEB protocol. Then NcoI-HF was heat-inactivated by incubating the reaction at 65°C for 20 min (Supplementary Figure S2). The single-site substrates 1 and 2 (SS 1 and SS 2) were obtained by double digesting the two-site substrate with NdeI and NcoI-HF followed by a 1% agarose gel extraction using the QIAquick^®^ extraction protocol (Supplementary Figure S2). The two-site substrate 2 (DS 2) was obtained by digesting a plasmid, also a derivative of the pHis17 vector, having five SauUSI sites with NdeI and EcoRV, followed by a 1% agarose gel extraction (Supplementary Figure S2). The 120 bp methylated, hemimethylated and non-methylated substrates were generated by an extension of two partially complementary 70 bp DNA oligos using a PCR machine (Supplementary Table S1).

### DNA cleavage assays

DNA cleavage assays were performed to test the nuclease activity of SauUSI. Initial cleavage assays were done using 150 ng of a circular plasmid. The substrate (plasmid) was incubated with 150 nM SauUSI and 2 mM ATP in the NEBuffer™ 4 (50 mM potassium acetate, 20 mM Tris-acetate, 10 mM magnesium acetate, 1 mM DTT pH 7.9) for 1 h at 37°C. Post incubation, the reaction was stopped by adding 1X Gel Loading Dye Purple and heating the reaction to 65°C for 20 min. The cleavage fragments were analyzed on a 1% agarose gel. Cleavage assays involving DS 1, SS 1 and TS were carried out with ∼3 nM DNA, 2 mM ATP and variable concentrations of the protein as mentioned in the respective figures. All other parameters were as mentioned above. Time-dependent assays of DS 1 and SS 1 were carried out across a time range.

Cleavage assays involving DS 2 and SS 2 were carried out with ∼10 nM DNA and 2 mM ATP across a concentration range of protein, which is mentioned in the respective figures. Cleavage products were resolved using a 2% agarose gel. 38 nM of the 120 bp methylated, hemimethylated and non-methylated substrates were used for cleavage by 100 nM SauUSI and 2 mM ATP and resolved on an 8% native PAGE. The position of the circular plasmid and the cleaved fragments were determined using LabImage^®^ (Kapelan Bio-imaging). Quantification of the intensities of the fragments from the DS 1, SS 1 and TS upon digestion was performed relative to the intensity of the corresponding fragments from a BstNI reaction. The BstNI reaction was performed to correct for the staining effect due to the variable length of the DNA fragments. The absolute intensities of the fragments obtained upon cleavage of the respective plasmids by BstNI were compared with the absolute intensity of the corresponding substrate (linearized plasmid) band. The ratio of the individual intensities so obtained was treated as the maximum intensity possible for a particular fragment. Next, a similar analysis was performed for DNA digested by SauUSI. The ratio of the relative intensity of a fragment obtained by SauUSI digestion with that of the relative intensity of the corresponding fragment obtained by BstNI digestion was calculated and plotted. The error bars represent the standard error of the mean across three independent assays. The error bars for the cleavage assay of SS 1 with 400 nM DNA represent the standard error of the mean across two independent assays. Cleaved products were analyzed using ImageJ, and their relative intensities were plotted using GraphPad Prism.

### Run-off sequencing

A 200 μl mixture containing 4 μg of a single-site substrate, 400 nM SauUSI and 2 mM ATP was incubated at 37°C till the reaction reached completion (checked on a 1% agarose gel). On completion of the reaction, the DNA fragments were purified using the QIAquick^®^ protocol. The purified fragments were sequenced (Sanger sequencing) using forward and reverse primers.

### SEC-MALS

1 mg/ml of the SauUSI was injected into a GE Superdex 200™ column equilibrated with 50 mM Tris–HCl, pH 8, 50 mM NaCl and 1 mM DTT. The apparatus was connected to a light scattering diode array and a differential refractive index detector (Wyatt Technology). The data was analyzed using the ASTRA software 6.1.7.17. The molar mass was calculated from the light scatter and differential refractive index. Before use, the column was calibrated using bovine serum albumin.

### Crystallization

To obtain phase information, we purified the Se-Met derivative of SauUSI (SauUSI^SelMet^). SauUSI^SelMet^ was crystallized by the hanging-drop vapor diffusion method using a 1:1 protein: reservoir buffer at 291K. The crystals were set in a 24-well plate with the reservoir buffer containing 0.1 M sodium citrate, 11–14% PEG 4000 and 0.25–0.4 M ammonium sulfate. Ethylene glycol was used as a cryoprotectant.

### X-ray data collection and processing

Crystals were first screened for diffraction quality using an in-house X-ray diffraction system of Rigaku MicroMax 007 X-ray generator with a Mar Research 345D detector. The crystals that diffracted best at home source were retrieved and taken for diffraction studies at synchrotron facilities at Diamond Light Source (DLS) Oxfordshire, UK, and European Synchrotron Research Facility (ESRF), Grenoble. The diffracted data was indexed and processed using XDS (25). The data sets were scaled and merged using AIMLESS (26).

### SAD phasing, structure solution and refinement

The structure of SauUSI^SelMet^ was solved using experimentally determined phases to 3.1 Å by single-wavelength anomalous dispersion (SAD) method (Supplementary Table S2). The selenium positions were identified using the program SOLVE-RESOLVE (27,28) in the Phenix suite of crystallography programs (29). In all, 37 seleniums were located in the asymmetric unit. A simple Fourier map calculated using the SAD phases was used to build the structure of SauUSI manually. Initial electron density maps were visualized, and further model building was carried out using COOT (30). Structure refinement was carried out using *phenix.refine* (31).

### DNA binding studies

The substrates used for the DNA binding study were generated using an annealing PCR (Supplementary Table S1). Different concentrations of SauUSI were allowed to incubate with the DNA on ice for 20 min. EMSA was carried out on a 5% native PAGE gel and was stained with ethidium bromide.

### Site-directed mutagenesis (Nuclease-inactive mutant)

An inactive nuclease point mutant, i.e. SauUSI^H119A^, was developed using the quick-change site-directed mutagenesis strategy. The mutation was confirmed by sequencing the plasmid. Purification of SauUSI^H119A^ was performed using the protocol described above.

### ATPase assay

The malachite green method was used to determine the ATPase stimulation in a DNA dependent and independent manner. 5 nM SauUSI^H119A^ was allowed to incubate with 2 mM ATP and 10 nM non-methylated DNA as well as, 2 mM ATP and 10 nM methylated DNA separately in a time-dependent manner. The inorganic phosphate released upon ATP hydrolysis formed a complex with molybdate and could be estimated colorimetrically at 630 nm. The results were compared to an appropriate phosphate standard curve to obtain inorganic phosphate release versus time in minutes and was fit using the one-phase association function in GraphPad Prism. The equation used was: *Y* = *Y*_max1_(1 – e^−*K*.*X*^). The error bars represent the standard error of the mean across three independent experiments.

### Triplex displacement assay

The substrate of the triplex displacement assay had two components: A Triplex Forming Oligo (TFO), 100 nM of which was labelled at the 5′ end using T4 polynucleotide kinase in the presence of ^32^P-γATP at 37°C for 30 min. Post the incubation, the reaction was stopped by heat inactivating T4 PNK at 65°C for 20 min. MicroSpin columns (GE) was used for purifying the TFO and stored at −30°C for future use. The second component was a plasmid DNA consisting of an enzyme binding cassette and a triplex binding site (TBS) 604 bp downstream of the enzyme binding cassette. Methylation and linearization of the plasmid were done with a similar protocol to the plasmids used for cleavage assays. To form the triplex, 50 nM of the linearized plasmid was incubated along with 25 nM TFO, 10 mM MES (pH 5.5) and 200 mM MgCl_2_ at 57°C for 15 min. Subsequently, the reaction was allowed to cool to 20°C and kept overnight. The triplex displacement reaction was carried out at 20°C at different time points by incubating SauUSI^H119A^ with the triplex DNA for 2 minutes following which cold TFO and ATP were added. The curve (% of triplex displaced versus time in minutes) was fit using the one-phase association function in GraphPad Prism. The equation used was: *Y* = *Y*_max1_(1 – e^−*K*.*X*^). The error bars represent the standard error of the mean across three independent experiments.

### Phenogram generation

The SauUSI primary sequence was used as a query for a pBLAST against REBASE and a tnBLAST in NCBI. Sequences above the 30% sequence identity were collated in JalView and aligned using Clustal Omega. Redundant sequences were removed by keeping the redundancy threshold at 90%. A primary tree was generated in JalView using the neighbor joining method. The tree was viewed using the iTOL software and rooted to SauUSI (32).

## RESULTS

### Cleavage products of SauUSI differ from those of site-specific cutters

On analyzing plasmid DNA treated with SauUSI and ATP, we observed that it appeared as a smear on an agarose gel (Figure 1A). Furthermore, the smearing was more pronounced as the number of target sites increased. For example, a plasmid DNA purified from *E. coli* (*dcm^+^*), which has five 5′-C^5m^CWGG-3′ sites, on treatment with SauUSI and ATP resulted in a smear with the peak intensity at ∼2300 bp (Figure 1A, Supplementary Figure S3a). The theoretical length of the largest fragment expected for SauUSI as a site-specific cutter is 2068 bp (Supplementary Figure S3b). The site-specific ATP-independent Type II REase BstNI, which recognized the same but non-methylated target site, i.e. 5′-CCWGG-3′, cut the unmodified plasmid into discreet fragments with the longest being ∼2200 bp (Figure 1A, Supplementary Figure S3c, lane 5).

This plasmid when purified from *E. coli* (*dcm^−^*) and treated with MspI methyltransferase (M.MspI) resulted in thirteen modified 5′-^5m^CCGG-3′ sites, ten of which were target sites of SauUSI (Supplementary Figure S3d). Treatment of the modified plasmid with SauUSI and ATP resulted in a smear with the peak at ∼530 bp (Supplementary Figure S3d). On the other hand, the expected length of the largest fragment was 657 bp if SauUSI was a single-site cutter (Supplementary Figure S3b). The unmodified plasmid, on incubation with the site-specific Type II REase MspI (R.MspI), which cuts at the target site 5′-CCGG-3′, resulted in discreet fragments with the longest being ∼510 bp (Supplementary Figure S3c, lane 3).

Next, incubating the plasmid purified from *E. coli* (*dcm^+^*) with M.MspI resulted in a 15-site SauUSI substrate, which upon cleavage resulted in a smear with the peak at ∼350 bp (Supplementary Figure S3d). In contrast, double digestion of the unmodified plasmid with the REases R.MspI (cut at thirteen 5′-^5m^CCGG-3′ sites) and BstNI (cut at five 5′-CCWGG-3′ sites) resulted in discreet fragments with the longest being ∼470 bp (Supplementary Figure S3c, lane 7). The expected size of the largest fragment if SauUSI was a site-specific cutter was 495 bp (Supplementary Figure S3b). Thus, the above analysis demonstrated that the cleavage product of SauUSI was different from a site-specific cutter, such as the Type II REases, which suggested that the enzyme had a distinct mechanism of DNA cleavage.

### SauUSI can nucleolytically cleave single-site DNA

To gain a better understanding of the nucleolytic activity of SauUSI, we focused our attention on a linear DNA substrate (DS 1) containing two target sites (5′-C^5m^CTGG-3′) separated by 1300 bp, which was generated by linearizing a 5034 bp plasmid DNA purified from *E. coli* (*dcm^+^*) (Figure 1B). It was previously proposed that SauUSI cleaves only that DNA that have at least two target sites and that the cleavage is close to one of the two target sites (12). Hence, we expected the SauUSI treated two-site substrate to yield fragments of length around 4394 bp and 1944 bp. However, a nuclease assay of the two-site substrate with enzyme concentration varying from 10 to 400 nM revealed three distinct bands corresponding to ∼3089, ∼1300 and ∼639 bp resulting from double-strand (ds) DNA breaks at or near both the target sites (Figure 1B). Additionally, less intense bands viz., 4394 bp and 1944 bp corresponding to DNA cleavage only at one of the two target sites were visible at lower concentrations of the enzyme (Figure 1B).

The disappearance of the 4394 and 1944 bp DNA with increasing concentration of SauUSI suggested that the cleavage of the two-site substrate happened in a step-wise manner, with the first cleavage event happening at one of the two target sites followed by cleavage at the second site. This was corroborated by a time-dependent cleavage assay carried out at an enzyme concentration of 100 nM, which showed diminished intensities of 4394 and 1944 bp DNA with increasing incubation time (Figure 1C). This result led us to investigate if SauUSI could cut a single-site substrate. For this, a 3627 bp linear DNA (SS 1) with a single SauUSI target site was generated (see Materials and Methods). The target site was flanked by 3089 and 533 bp DNA (Figure 1D). On performing a SauUSI concentration-dependent DNA cleavage assay, we found that the DNA was cut close to the target site (Figure 1D), albeit with an efficiency lower than that noted for the two-site substrate (Figure 1E, F).

### SauUSI cuts DNA at multiple locations away from the target site

**Figure 2.**
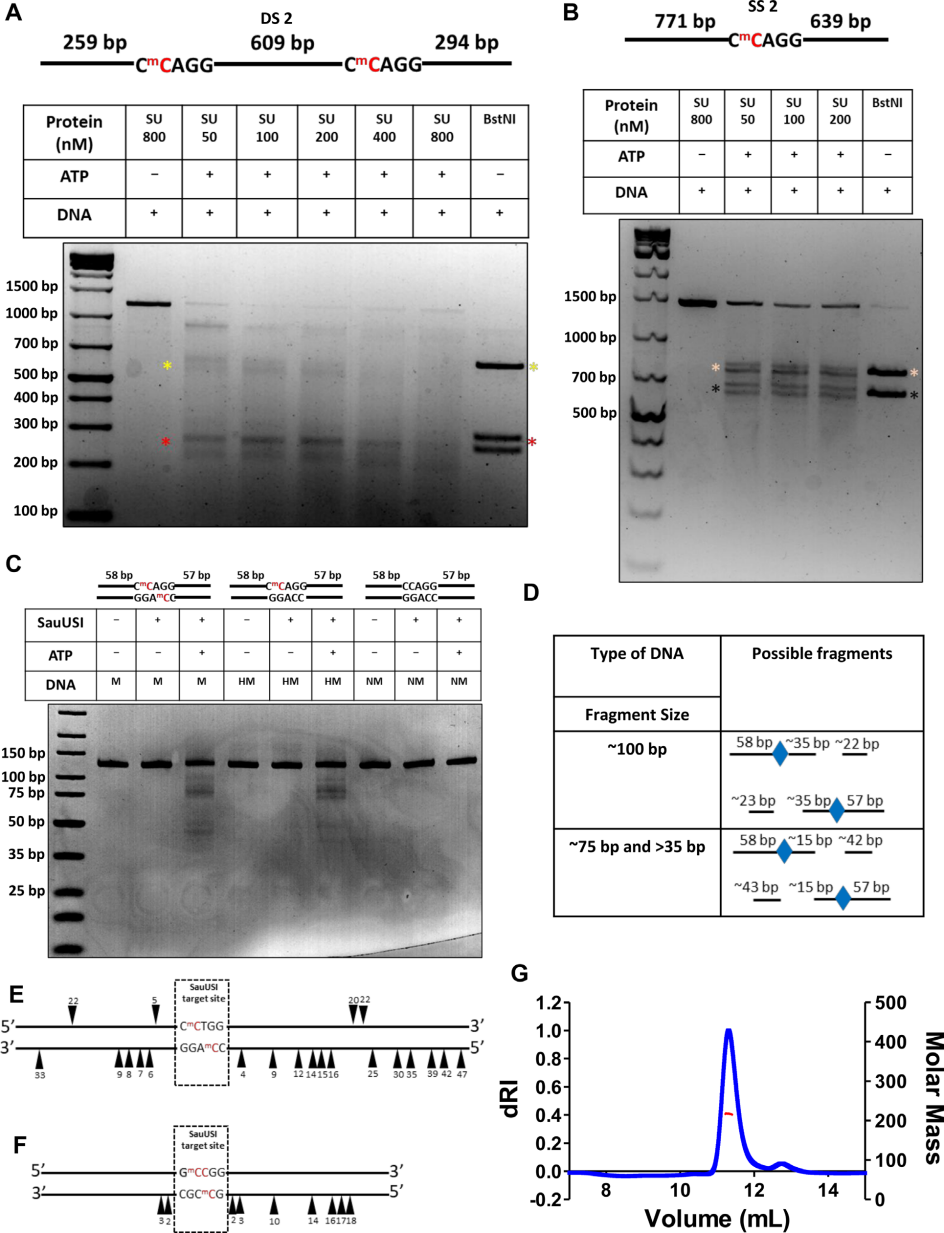
Position of cleavage by SauUSI and oligomeric status of SauUSI. (**A**) A two-site substrate (DS 2) used for the cleavage assay; the separation between the two sites is 609 bp. The cytosine highlighted in red represents methylation. The target site is methylated on both the strands. Representative 2% agarose gel for the two-site cleavage using varying concentrations (50–800 nM) of SauUSI. The yellow and red asterisks represent the central (∼609 bp upon BstNI treatment) and flanking regions (∼294 and ∼259 bp upon BstNI treatment) of the DNA post cleavage, respectively. (**B**) A single-site substrate (SS 2) used for the cleavage assay. The cytosine highlighted in red represents methylation. The target site is methylated on both the strands. Representative 2% agarose gel for the single-site cleavage using varying concentrations (50–200 nM) of SauUSI. The beige and black asterisks represent the two fragments (∼771 and ∼639 bp upon BstNI treatment) of the DNA post cleavage. (**C**) 120 bp methylated, hemimethylated and non-methylated substrates used for the cleavage assay. The cytosine highlighted in red represents methylation. Representative 8% Native PAGE gel for the 120 bp substrates. (**D**) Interpretation of the possible cleavage products from panel C. (**E**) Cartoon representation of the sequencing of the single-site DNA after cleavage by SauUSI. The arrowheads represent nicks on the DNA substrate, the position of the arrowheads illustrates on which strand of the DNA the nicks were observed and how far away they are, in base pairs, from the target site. (**F**) Cartoon representation of the nicks on a two-/multi-site substrate after cleavage by SauUSI identified using run-off sequencing (data from Xu *et al.*, 2011). (**G**) SEC-MALS chromatogram of SauUSI shows the refractive index signal with the derived molar mass indicated by a red horizontal line.

Previously, it was reported that SauUSI causes multiple nicks close to the target site (12), which led us to find the locations of cleavage. To identify the location of cleavage, we carried out the following experiments. We performed a cleavage assay using SauUSI and BstNI on a two-site substrate (DS 2) which had the sites spaced 609 bp apart and visualized them on an agarose gel (Figure 2A). It was evident that the fragments generated upon treatment with SauUSI migrated slightly lower than that of the fragments generated by BstNI (Figure 2A). This indicated that SauUSI cleaved the DNA away from the target site. Furthermore, when we treated a 1415 bp single-site substrate (SS 2) with SauUSI, a pair of doublets were visible on the gel about the size of the fragments generated by the single-site cutter BstNI (Figure 2B). This suggested that SauUSI made multiple dsDNA breaks beyond the target site.

To better resolve the fragments generated by SauUSI cleavage at multiple points, we used a 120 bp substrate with a target site flanked by 57 and 58 bp DNA on either side (Figure 2C). Multiple fragments were observed on the cleavage of the 120 bp methylated DNA by SauUSI (Figure 2C). An eyeball estimation of the sizes of the fragments indicated three distinct types: fragments that were ∼100 bp, which would form if cleavage occurred ∼35 bp away from the target site; fragments that were ∼75 and >35 bp, which would form if cleavage occurred ∼15 bp away from the target site (Figure 2D). The fragments that are ∼75 and >35 bp seem to be of greater intensity than the ∼100 bp fragment, which indicates that dsDNA breaks occur predominantly ∼15–20 bp away from the target site.

Additionally, we studied the cleavage of a 120 bp hemi-methylated single-site substrate (Figure 2C). The cleavage pattern was similar to that of the methylated DNA but not the same, possibly because the enzyme could bind to the target sites in only a single orientation, unlike the two orientations possible in case of a palindromic fully methylated site. However, the cleavage efficiency of both the substrates was low, indicating the requirement of a minimum substrate length for efficient cleavage. Single-site cleavage was not observed previously, possibly because of the comparatively short length of the DNA used for the assay (12). As expected, a 120 bp DNA with non-methylated target sequence was not cleaved by SauUSI (Figure 2C).

Cleavage of a single-site substrate (SS 1) was used to map the location of the nicks causing the dsDNA breaks using run-off sequencing. The sequencing was performed both in the forward and reverse directions to locate nicks on the lower strand and upper strand of the DNA, respectively. Sequencing in the forward direction located nicks 4, 9, 12, 14, 15, 16, 25, 30, 35, 39, 42 and 47 bp to the right-hand side (RHS) and 6, 7, 8, 9 and 33 bp to the left-hand side (LHS) of the target site (Figure 2E, Supplementary Figure S4). Sequencing in the reverse direction also showed multiple nicks on the RHS (20 and 22 bp) and LHS (5 and 22 bp) of the target site (Figure 2E, Supplementary Figure S5). The presence of nicks ∼15–20 bp away from the target site corroborated with the cleavage data obtained from the 120 bp methylated substrate. However, since the efficiency of cleavage for the 120 bp substrate is low, the optimal range of cleavage may differ for longer substrates, which are cut more efficiently by SauUSI.

The noise in the sequencing data increased at >45 bp away from the target site, thereby making it difficult to interpret if there were nicks beyond this distance. The position of the nicks that we observed were consistent with the location of the cleavage mapped by Xu *et al.* (12) from a two-site substrate and a multi-site substrate (Figure 2F). The small variation in the location of cleavage reported by the two studies could be a result of the difference in the sequence of the DNA used, which can affect the exact location of cleavage. Effect of sequence on the location of cleavage has been reported previously in the case of Type ISP REases (22).

### SauUSI exists as a dimer in solution

dsDNA break requires cleavage of at least two spatially close phosphodiester bonds, one each from the two strands of the duplex. Usually, an endonuclease catalyzes DNA cleavage by one of the following means. A constitutive homodimeric enzyme binds to the target site, and each of the two nuclease active sites cleaves one of the two phosphodiester bonds, as in the case of many Type II REases (33); or the two active sites of a monomeric enzyme cleave the two phosphodiester bonds as in homing endonuclease PI-*Sce*I (34); or a single active site formed by a dimeric endonuclease cuts the strands sequentially as in the Type II REase BfiI (35); or two monomeric enzymes associate transiently to catalyze the cleavage of the two neighboring phosphodiester bonds as in the case of the REase FokI or the Type III RM enzymes (23,36); or the two nuclease domains come together in *cis* by translocation along the DNA as in the case of Type I and Type ISP RM enzymes (22,37). Thus, an analysis of the oligomeric structure of the enzyme can provide insights into the mechanism of DNA cleavage. We characterized the oligomeric structure of SauUSI using size exclusion chromatography coupled with multi-angle light scattering (SEC-MALS). A clear monodisperse peak was observed from the SEC-MALS run (Figure 2G). The scatter at 659 nm corresponded to a mean molecular weight (*M*_avg_) of 217.7 (±0.022%) kDa, which is approximately twice the theoretical mass of the monomeric protein (109.9 kDa). Therefore, we concluded that SauUSI existed as a dimer in solution.

### Molecular architecture of SauUSI

**Figure 3.**
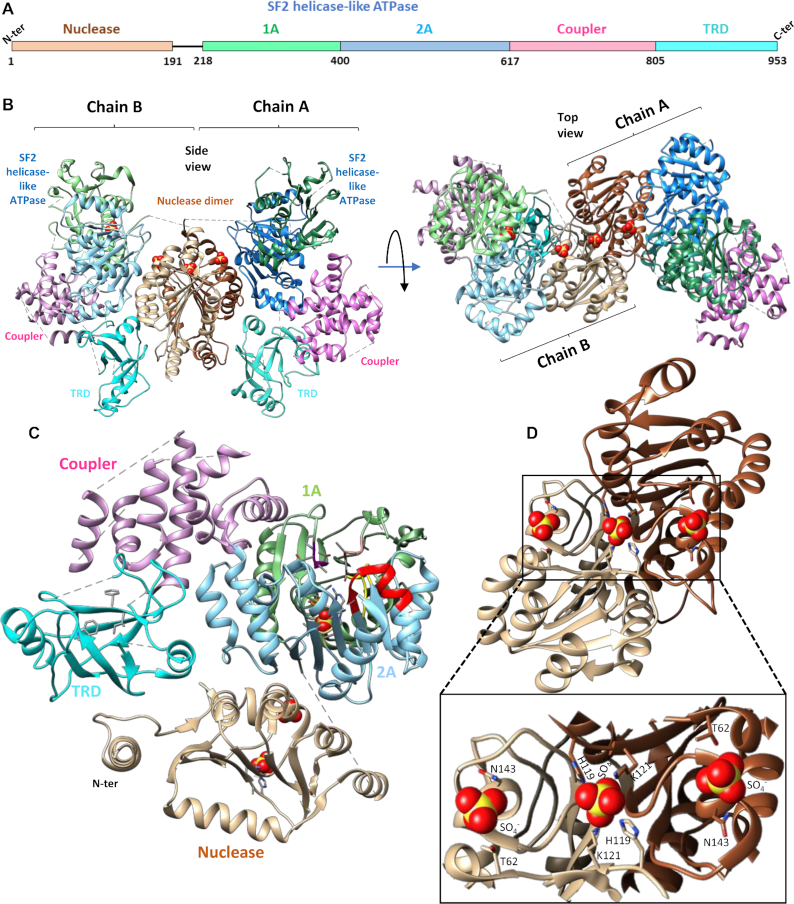
Molecular architecture of SauUSI. (**A**) The primary domain arrangement in a SauUSI protomer, the line connecting the nuclease domain to the SF2 helicase-like ATPase signifies an unstructured linker. (**B**) Ribbon diagram of two views of the crystal structure of the dimeric SauUSI. Each structural domain of the protomers is colored distinctly. The sulfates are represented as spheres. (**C**) Structure of a protomer of SauUSI with nuclease domain in tan; the 1A and 2A domains of the ATPase having the RecA fold in green and blue, respectively; the coupler domain in pink; the TRD (SRA) domain in cyan. Certain important motifs/residues of domains are highlighted in sticks. (**D**) Structure of the nuclease from the two protomers highlighting the dimeric interface. A zoomed view of the DNA binding region of the nuclease dimer identified using the bound sulfate ions shown as spheres.

The homodimeric assembly of SauUSI suggested that the enzyme could perform dsDNA breaks by employing two nuclease active sites to catalyze the hydrolysis of the phosphodiester bonds on either strand. Usually, such dimeric endonucleases have the oligomeric interface formed by the nuclease domains, with their respective active sites being spatially close to each other (38). To ascertain the architecture of SauUSI, we crystallized the apo-enzyme and determined the structure using X-ray crystallography at a resolution of 3.1 Å (Supplementary Table S2). The structure revealed that the single polypeptide protein SauUSI is a dimer and is made of four domains—the N-terminal nuclease domain, followed by a Superfamily 2 (SF2) helicase-like ATPase domain, and an α-helical coupler domain that connects the ATPase to the target recognition domain (TRD) (Figure 3A–C). The structure also revealed that the interaction of the nuclease domains of the respective monomers stabilized the dimeric assembly (Figure 3B and D).

The SauUSI nuclease domain (residues 1–191) belongs to the phospholipase D (PLD) family nucleases with the conserved 119-H(X)K(X)4E-126 motif (Supplementary Figure S6a). The dimeric interface of SauUSI formed by the two nuclease domains had a buried surface area of 1970 Å^2^ and was reminiscent of the interface seen in the crystal structure of the PLD family nuclease BfiI (PDB ID: 2C1L) (Figure 3B and D, Supplementary Figure S6b) (39). In the dimeric assembly of SauUSI, the nuclease catalytic site appeared pre-formed by the ‘HxK’ motifs from the two protomers. Three sulfate ions, which possibly mimic the phosphate backbone of a substrate DNA, highlighted the path that the DNA would take on binding to the nuclease domain (Figure 3B and D). One of them was bound to the active site residues His-119 and Lys-121 of both the protomers, while the other two interacted with the main chain of Ser-142, and the side chains of Thr-62 and Asn-143 from the two protomers, respectively. Sulfate ions were part of the crystallization condition.

Experiments on BfiI, a Type II REase with a PLD nuclease domain, have shown that the single active site (formed by the two protomers) cuts both the strands of DNA by undergoing a 180° conformational switch and that both the active site histidines (from the two protomers) are not functionally equivalent. Instead, the roles of the histidines are sequential. One of the histidines functions as a nucleophile that attacks the phosphate backbone and forms a histidine-phosphate intermediate. Then this covalent intermediate is broken with the help of the second histidine (40). However, a similar mechanism of DNA cleavage by SauUSI remains to be verified.

The nuclease domain of SauUSI is connected to the ATPase domain by a long linker (residues 192–220). The linker could not be built as the corresponding electron density was absent. In the case of BfiI, it is hypothesized that the linker between the PLD nuclease and TRD plays a role in repressing nucleolytic activity of the PLD domain by mimicking the nucleic acid backbone (39). The linker in SauUSI is 26 amino acids long and possess negatively charged residues Glu-194, Glu-199, Glu-203, Glu-205 and Asp-213. Alternatively, the linker could structurally hinder the accessibility of the nuclease active site thereby potentially acting as a regulator of the nuclease activity. If and how the linker regulates the nucleolytic activity of SauUSI need to be further examined.

The RM systems CglI and NgoAVII also possess PLD nucleases and SF2 helicase-like ATPases (41) (Supplementary Figure S7a and S7b). However, unlike SauUSI, NgoAVII and CglI are multi-subunit enzymes having R_2_H_2_ stoichiometry, where R and H are the two subunits. The R-subunit consists of the PLD nuclease domain and B3 TRD whereas, the H-subunit consists of the ATPase domain along with the accessory domains Z1 and C. Though, NgoAVII/CglI contains a PLD nuclease and an SF2 helicase-like ATPase, the differences in their domain arrangement from that of SauUSI hint at a distinct mode of DNA cleavage in the two types of enzymes.

### Recognition of the methylated target sequence by the TRD activates the ATPase

**Figure 4.**
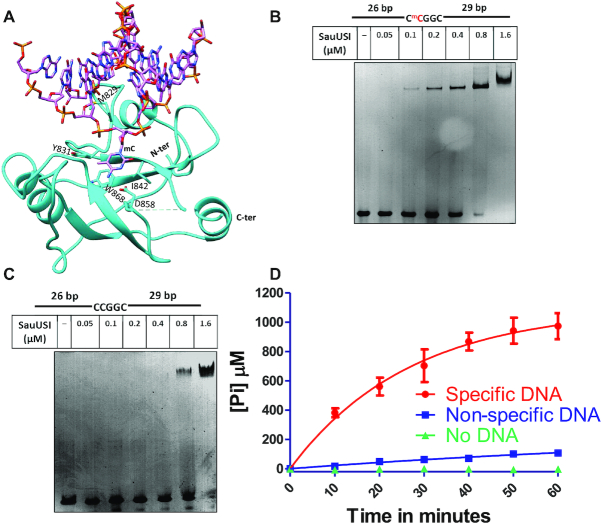
Target recognition by the SRA domain and DNA dependent ATP stimulation of SauUSI. (**A**) Structure of the SRA domain of SauUSI with DNA modeled. The DNA is from the structure of the structural homolog SUVH6, an H3K9 histone methyltransferase from *Arabidopsis thaliana* (PDB ID: 6A5N). (**B**) The specific DNA substrate used for EMSA. The cytosine highlighted in red represents methylation. The target site is methylated on both the strands. Representative 5% native PAGE EMSA gel using specific DNA substrate (250 nM) and assay performed over a protein concentration range of 0.05–1.6 μM. (**C**) The non-specific DNA substrate used for EMSA. Representative 5% native PAGE EMSA gel using non-specific DNA substrate (250 nM) and assay performed over a protein concentration range of 0.05–1.6 μM. (**D**) ATPase stimulation assay carried out using the malachite green method, represented by inorganic phosphate released (on the Y-axis) over time (on the X-axis) in reactions containing specific DNA, non-specific DNA and no DNA (*n* = 3).

At the C-terminus of the protein is the TRD (residues 806–953), which is linked to the ATPase domain by the coupler domain (residues 618–805). The structure of the TRD revealed that it has the fold of an SRA (SET and RING associated) domain, a fold that specializes in recognition of 5-methylcytosine in DNA (Figure 4A). A search using the DALI server (42) identified the SRA domain of SuvH6 from *Arabidopsis thaliana* (PDB ID: 6A5N) (43), a histone methyltransferase, which belongs to the SuvH family, to be the closest structural homologue of the SauUSI SRA domain (Supplementary Figure S8a). Based on the DNA-bound structure of SuvH6 and the corresponding structure-based sequence alignment (Supplementary Figure S8b), a model of SauUSI SRA domain bound to the target sequence was generated (Figure 4A).

The model predicted SauUSI SRA to approach a DNA duplex from its minor groove side and flip out the 5-methylcytosine of the target sequence into a hydrophobic pocket lined by the residues Tyr-831, Phe-841, Ile-842 and Trp-868, while Met-829 inserted into the cavity formed in the DNA by base flipping (Figure 4A). Asp-858 is positioned to make base-specific hydrogen bonds with the flipped cytosine, Tyr-831 is positioned to interact with the methyl group to establish specificity for the methylated cytosine, and Trp-868 could stack against and stabilize the flipped base. Additionally, the region between 848 and 854 is disordered in the apo-structure, which may interact with the flipped base upon DNA binding, as predicted based on the role of the structurally equivalent loop in SuvH6. SauUSI can also recognize target sequences having 5-hydroxymethylcytosine (5hmC) and cleave such DNA (12). Studies on SuvH5, another close homolog of the SRA domain of SauUSI, indicate that the mechanism of recognition of 5-methylcytosine and 5hmC is similar, and the binding affinities are comparable (44). We hypothesize a similar situation for the SRA domain of SauUSI.

Base-specific interaction with 5-methylcytosine in the target sequence was found to be essential for SauUSI to discriminate between a specific DNA substrate and a nonspecific DNA. Replacement of 5-methylcytosine by cytosine in the recognition sequence reduced the affinity of SauUSI for the DNA as observed by electrophoretic mobility shift assay (EMSA) using a 60 bp DNA (Figure 4B and C). A shift in the DNA was detected at 0.1 μM of SauUSI for specific DNA, whereas for non-specific DNA it was at 0.8 μM. Furthermore, the ATPase activity of SauUSI was stimulated on recognition of the target sequence (Figure 4D). An inactive nuclease point mutant, SauUSI^H119A^, was used for studying the ATPase activity of the enzyme to prevent cleavage of the DNA in the assay. We found that the ATPase activity of 5 nM of SauUSI^H119A^ incubated with 2 mM ATP was negligible in the absence of DNA as measured by quantifying the phosphate released using the malachite green ATPase assay. Addition of 10 nM non-specific DNA (in which the target sites were not methylated) resulted in only a marginal increase in the activity. However, in the presence of 10 nM specific DNA, a significant amount of ATP was hydrolyzed. Since the enzyme used was an inactive nuclease point mutant of SauUSI, there is a possibility of multiple rebinding events of the enzyme to the substrate. Hence, the amount of phosphate released cannot be directly compared to the cleavage rates.

The manifold increase in the ATPase activity of the enzyme upon recognition of a specific DNA could be a result of two possible steps occurring sequentially. One, the SRA domain's intrinsic preference to bind methylated DNA over non-methylated DNA, thereby leading to the engagement of the ATPase with only specific DNA. Two, activation of the ATPase upon binding to a specific DNA, which results in conformational changes transduced from the target-sequence-bound SRA domain to the ATPase and due to the binding of the ATPase to DNA. The coupler domain that connects these two domains may play a role in this allostery. The coupler was made of two subdomains: a subdomain of four short helices (618–669) and another made of at least six helices (673–805). The electron density corresponding to residues 710–773 of the coupler domain was poor, and the region could only be built partially.

### The ATPase powers DNA translocation by SauUSI

**Figure 5.**
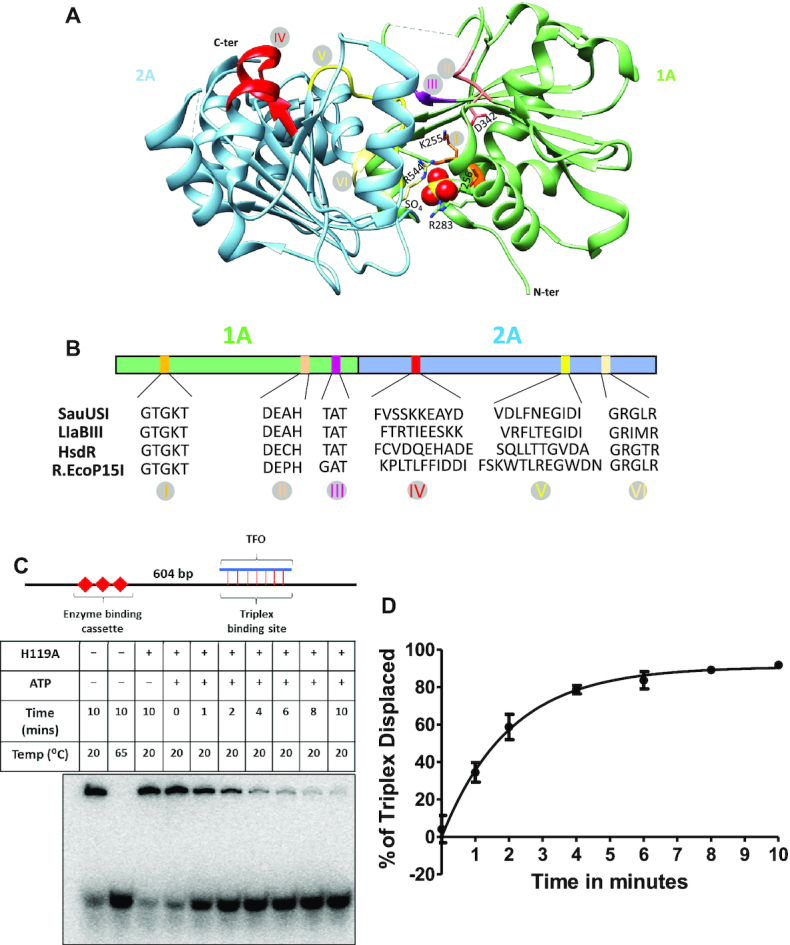
ATPase-driven DNA translocation by SauUSI. (**A**) The primary domain architecture of the SF2-helicase like ATPase along with the canonical motifs marked in specific colors. The canonical motifs of the SF2-helicase like ATPase of SauUSI are compared to that of LlaBIII (Type ISP REase), HsdR (Type I REase) and EcoP15I (Type III REase). (**B**) Structure of the ATPase domain with the two RecA folds colored distinctly. The catalytically important Walker A and Walker B residues, along with a sulfate ion (shown as a sphere) bound at the ATP binding site are shown. Also, illustrated are the other canonical motifs in the same color code as in panel A. (**C**) The DNA used for the triplex displacement assay has an enzyme binding cassette located 604 bp away from the triplex binding site. A representative PAGE gel of the triplex displacement assay carried out over a time period of 1–10 min. (**D**) Translocase activity of SauUSI^H119A^ monitored by the percentage of triplex displaced as a function of time (*n* = 3).

The SauUSI ATPase belongs to the SF2 DExH helicase family. In the apo-structure of SauUSI, the two ATPases from each monomer are spatially separate and do not interact with each other. The SF2 helicase-like ATPase domain features two RecA-like sub-domains, i.e. 1A and 2A (residues 221–400 and 401–617) and the ATP binding pocket is located in a cleft between them, which in the crystal structure of the apo-enzyme was occupied by a sulfate ion (Figure 5A). A search for structural homologs of the ATPase of SauUSI using DALI server (42) revealed it to be similar to the ATPase domains of HsdR of the Type I REase from *Vibrio vulnificus* (PDB ID: 3H1T) and the Type ISP REase LlaBIII (PDB ID: 4XQK) (22,45). The 1A and 2A sub-domains of the SauUSI ATPase structurally aligned well with the corresponding sub-domains of LlaBIII and HsdR (Supplementary Figure S9). LlaBIII has a β-hairpin loop extending from the 1A sub-domain, which is essential for DNA translocation (46). SauUSI had a short-disordered loop at the structurally equivalent position, which may have a role in DNA interaction and translocation.

A sequence alignment of the ATPase of SauUSI with the corresponding domain in LlaBIII, HsdR and EcoP15I revealed the six conserved canonical motifs distributed amongst the two RecA-like sub-domains (Figure 5b). Motif I (Walker A) and motif VI (arginine finger) are involved in ATP binding, whereas motif II (Walker B) is involved in ATP hydrolysis. The nucleic acid interacting module of the SF2 helicase-like ATPase involves motif IV and motif V. Motif III helps in coupling ATP hydrolysis to nucleic acid translocation. The sulfate ion interacts with Thr-256 of the Walker A motif, Arg-283 and Arg-544 of motif VI (Figure 5A).

Previously, it has been shown that ATP hydrolysis is essential for activation of the SauUSI nuclease (12). The fact that SauUSI cut DNA close to one of the two target sites and that it could cut DNA having a single target site was reminiscent of the Type III REase. In a Type III REase, the ATPase functions as a switch to conformationally activate the enzyme upon ATP hydrolysis to perform long-range diffusion along the DNA and/or cleave it (23,24). Interestingly, the same family of ATPase in Type I and Type ISP REases function as dsDNA translocating motor that actively translocates the enzyme along the DNA. The convergence of two translocating enzymes results in DNA cleavage at the point of the meeting, which is random and located somewhere between two target sites (22,37).

To find out if the SauUSI ATPase, which is homologous to the ATPases of both Type I/ISP and Type III RM enzymes, functioned as a switch or as a motor, we performed a triplex displacement assay. The assay monitors the ability of an enzyme to displace a TFO (triplex forming oligo) from a DNA (47). A translocating ATPase motor can displace the TFO from the DNA, while an ATPase switch cannot displace the TFO (48). For the assay, we used a substrate DNA having a SauUSI binding cassette containing three closely spaced target sites and a TFO binding site 604 bp downstream of the cassette. The TFO was radiolabeled to visualize them on a non-denaturing polyacrylamide gel (PAGE). SauUSI^H119A^ was used for the assay to prevent DNA cleavage. Displacement of the TFO, if any, by 30 nM SauUSI^H119A^ was monitored as a function of time (Figure 5C and D). It was observed that in the presence of ATP, over 85% of the TFO was displaced within 10 min of the reaction (Figure 5D), indicative of DNA translocation by SauUSI. Displacement of the TFO was not observed in the absence of ATP.

**Figure 6.**
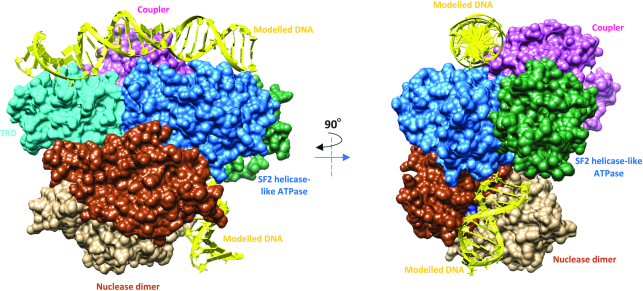
A hypothetical model of SauUSI bound to DNA. DNA modeled onto the TRD, ATPase and the nuclease domains of SauUSI. From the figure it is evident that the predicted path of the DNA on the nuclease domain (modelled based on the sulfate ions bound to the active site) is not aligned with the path of the DNA on the TRD and ATPase domains. Either the DNA must wrap around and interact with the nuclease active site or a major conformational change must be brought about in the protein for the nuclease active site to align along the path of the DNA bound to the TRD and ATPase.

We modelled a DNA into the target recognition domain and the SF2 helicase-like ATPase by structurally aligning two close homologs (TRD of SuvH6 and the SF2 helicase-like ATPase of LlaBIII) and noticed that the orientation of the TRD and the SF2 helicase-like ATPase was such that its DNA binding surface faced away from the expected path of the nuclease-bound DNA (as predicted by the position of sulfates). It was apparent that only a large conformational change could engage both the ATPase and the nuclease with the DNA simultaneously unless the DNA takes a convoluted path that wraps around the enzyme (Figure 6). Hence, the apo-structure of SauUSI may be that of an inactive state. Target recognition and ATP hydrolysis are steps towards the activation of the SauUSI nuclease.

### SauUSI nucleolytically shreds DNA having target sites at both its ends

**Figure 7.**
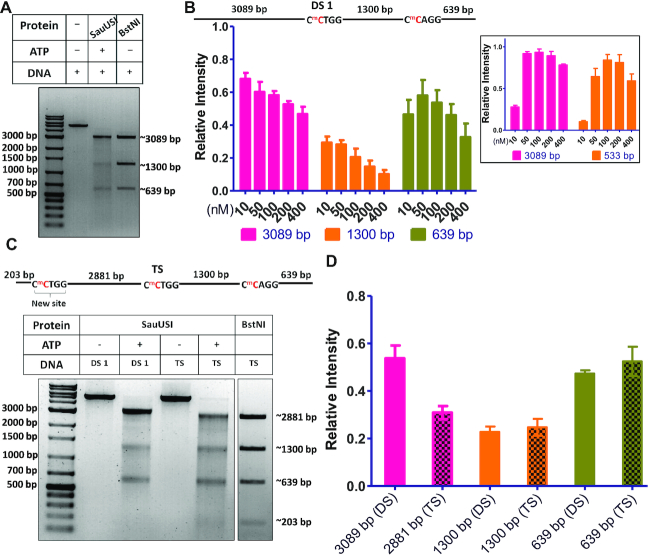
Reduced intensity of major fragments. (**A**) Two-site (DS 1) cleavage pattern comparison between SauUSI (100 nM) and BstNI (Type II REase) using 3 nM DNA at 37°C. (**B**) Schematic of the two-site substrate. The cytosine highlighted in red represents methylation. The target site is methylated on both the strands. Quantification (relative intensity) of the three major cleavage fragments of the two-site substrate across the concentration gradient of SauUSI, assay conditions as discussed in Figure 1B (*n* = 3). (*Inset*) The quantification (relative intensity) of the two major cleavage fragments of the single-site substrate (SS 1) across varying concentrations of SauUSI, as shown in Figure 1C (*n* = 2). (**C**) The three-site substrate used for the cleavage assay using 3 nM DNA and 100 nM SauUSI incubated at 37°C for 1 h. The cytosine highlighted in red represents methylation. Representative gel of the three-site substrate (TS) cleavage pattern in comparison to the cleavage seen with a two-site substrate (DS 1). For comparison, the product of the three-site substrate on cleavage with the REase BstNI is also shown. (**D**) The quantification (relative intensity) of the major cleavage products between the two-site substrate (DS 1) cleavage pattern and the three-site substrate (TS) cleavage pattern (*n* = 3).

Like Type I or ISP RM enzymes, SauUSI translocated DNA, however, unlike the former, SauUSI cut DNA close to one of the two target sites rather than somewhere in between the target sites. Furthermore, unlike Type I or ISP REases, SauUSI was able to cut linear DNA having a single target site (see above). These observations implied that though ATP hydrolysis was essential for the nucleolytic activity of SauUSI, the convergence of two such enzymes by DNA translocation was not essential. As discussed above, the mode of DNA cleavage by SauUSI appeared distinct from canonical Type I/ISP, II or III REases. This deduction was further strengthened when a careful examination of the cleavage product of the two-site substrate (DS 1) by SauUSI on an agarose gel revealed that of the three primary fragments, the intensity of the central ∼1300 bp fragment was disproportionately lower than the ∼3089 kb and ∼639 bp fragments that flank the two target sites, respectively. This was clear when the cleavage pattern of the two-site substrate by SauUSI was compared with the cleavage pattern of the same DNA, obtained from treatment with the Type II REase BstNI, which cuts at 5′-CC/^5m^CTGG-3′, the methylated version of which is the target site for SauUSI (Figure 7A).

The reduction in the intensity of the ∼1300 bp fragment became more pronounced with increasing concentration of SauUSI and was close to zero at 400 nM of enzyme concentration (Figure 7B). In contrast, the reduction in the intensity of the ∼3089 and ∼639 bp fragments were much less. Similarly, neither of the two DNA fragments generated by the single-site cleavage by SauUSI showed a disproportionate decrease in intensity (inset Figure 7b). This led us to hypothesize that the DNA between two target sites underwent further nucleolytic processing.

To test this hypothesis, we converted the two-site substrate to a three-site substrate (TS) by introducing a new site 203 bp away from one end of the DNA (Figure 7C). A comparative cleavage assay performed using the two-site substrate (3 nM) and the three-site substrate (3 nM) with 100 nM SauUSI and 2 mM ATP showed that there was a sharp decrease in the intensity of the ∼2881 bp fragment formed by the processing of the three-site substrate by SauUSI (Figure 7C and D). This was very similar to the decrease observed in case of the ∼1300 bp fragment of the two-site substrate. This assay confirmed that the DNA between two target sites underwent further nucleolytic processing. A similar trend was observed with the substrates DS 2 (Figure 2A). As the nucleolytic activity of SauUSI resulted in a smear when visualized on an agarose gel, which extended to almost the bottom of the gel, we concluded that the DNA was cut at multiple locations along the entire length of the DNA resulting in shredding. In comparison, DNA flanking the two target sites showed minimal smearing, suggesting that they were, if at all, minimally shred.

## DISCUSSION

The biochemical and structural analysis of SauUSI, a major barrier to HGT in *S. aureus*, revealed that the overall architecture of the enzyme and its mode of DNA cleavage were different from those of a *bona fide* Type I, ISP, II or III REases. Structural and SEC-MALS studies revealed that the single polypeptide chain of SauUSI is made of the nuclease, the ATPase, the coupler and the TRD, and exists as a homodimer in solution. The linear domain arrangement in SauUSI is similar to that of the ATP-independent Type II REase MmeI (lacking the ATPase domain) and the ATP-dependent Type ISP REases, both of which have a methyltransferase domain as well (Supplementary Figure S10) (22,49). However, the latter two, unlike SauUSI, are monomeric. We think that only one of the two TRDs of SauUSI will recognize and bind to the palindromic target site 5′-S^5m^CNGS-3′, enabling SauUSI to assemble on the target site in two different directions (Supplementary Figure S11) (50,51). Also, it is not yet clear if the enzyme orientation is random or directed by asymmetry in either the target site sequence or the target site methylation. Binding of the dimeric enzyme to the target site allosterically activates the ATPase domain, which was experimentally observed as stimulation of ATP hydrolysis upon addition of substrate DNA.

**Figure 8.**
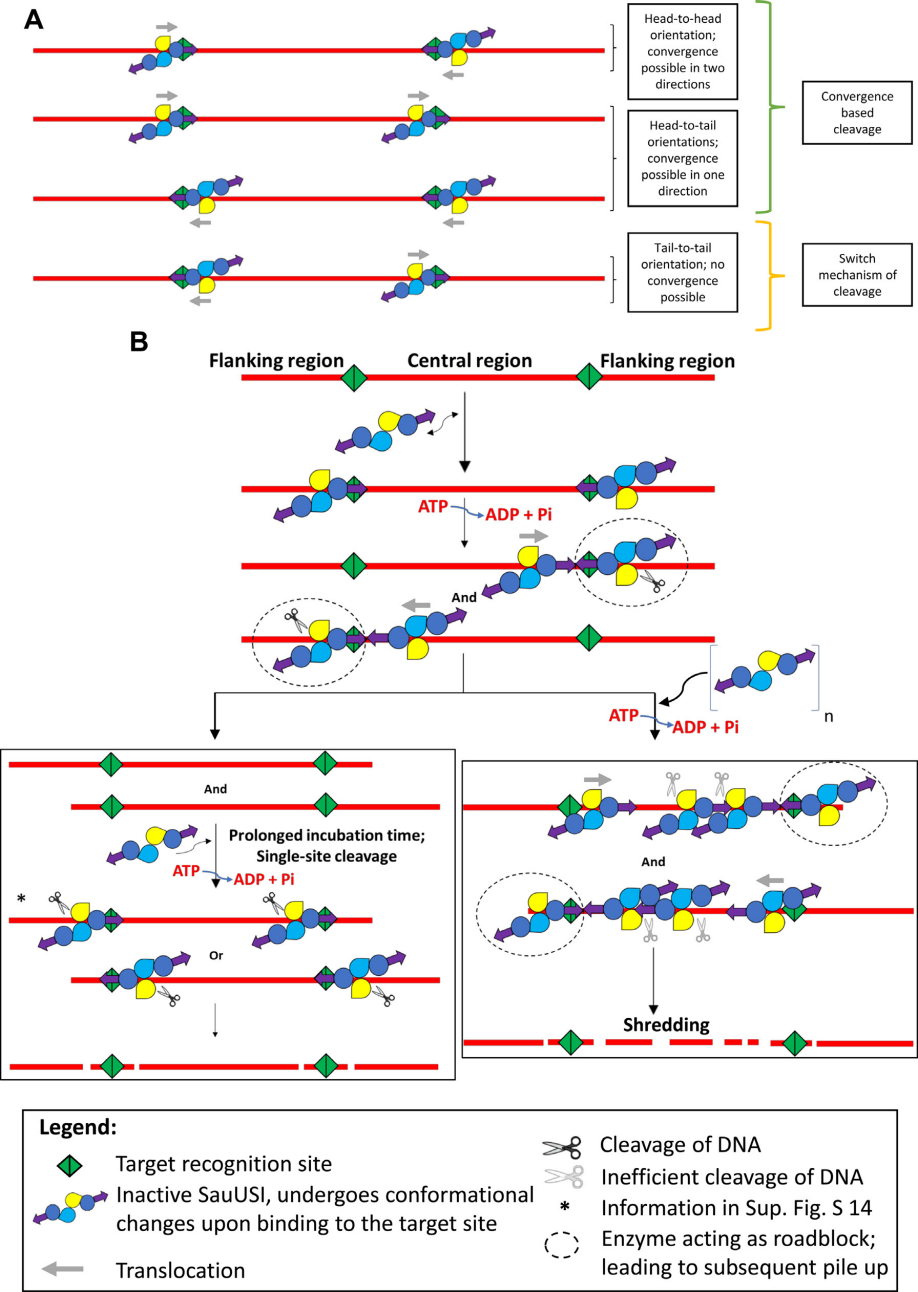
Model for the cleavage by SauUSI. (**A**) The different orientations that SauUSI can bind when there are two target sites. The head-to-head and head-to-tail orientations lead to possible convergence events leading to DNA cleavage. In the tail-to-tail orientation, however, there is no convergence event possible; therefore, it is cleaved by the switch mechanism (similar to single-site cleavage). Even though the probability of binding to each of these orientations is equal, cleavage seems to occur more rapidly when there is a possibility of enzymes converging. (**B**) A cartoon representing the model of cleavage of a two-site substrate when SauUSI molecules are bound in the head-to-head orientation. SauUSI is a homodimer with the interface at the nuclease domain (yellow and blue colors representing the nuclease domains). Each monomer has one helicase domain (dark blue circle) and one TRD (purple arrow). The enzyme binds to the DNA (red) possessing the target sites (green) undergoing a conformational change (represented by a slight change in the orientation in the cartoon). Upon ATP hydrolysis, a translocating SauUSI can converge with a stationary SauUSI (roadblock; black dotted circle around the roadblock) bound to another target site. The convergence stalls the translocating enzyme, and DNA is cleaved by the stationary enzyme (black pair of scissors). There are two routes that the cleavage can proceed depending on the concentration of the enzyme. When SauUSI is present in a limited concentration, the preceding convergence event leads to cleavage producing two two-site fragments. These fragments, upon prolonged incubation with SauUSI and ATP, undergo cleavage due to the switch activity of the ATPase eventually leading to discreet bands. However, when SauUSI is present in excess, then, there is multiple binding and translocation of SauUSI in either direction leading to a pile-up of enzymes along the central region. This, in turn, leads to the stalling of multiple enzymes in the central region. Inefficient nucleolytic activity (grey pair of scissors) of the stalled enzymes leads to random dsDNA breaks, thus shredding the central DNA fragment. Starred steps represent single-site cleavage events (refer to Supplementary Figure S13 for possible products formed).

Hence, on a two-site substrate, there are four possible arrangements of SauUSI on the DNA, i.e. head-to-head, head-to-tail, tail-to-tail and tail-to-head (Figure 8A). For the sake of simplicity, we shall focus on one of these possibilities, i.e. the head-to-head orientation (Figure 8B). As demonstrated using the triplex displacement assay, upon ATP hydrolysis SauUSI would translocate along the DNA. The translocating enzyme eventually converges in *cis* on to a stationary SauUSI molecule bound to the second target site. We propose that the stationary enzyme acts as a roadblock stalling the translocating enzyme, and predict that the physical convergence of the two enzymes stimulates the nuclease activity of the stationary enzyme resulting in dsDNA break. Our prediction of the stimulation of the nuclease upon convergence derives from the observation that a two-/multi-site substrate is cleaved with higher efficiency than a single-site substrate. Note that the convergence of two translocating SauUSI molecules, i.e. enzymes that have left their respective target sites, does not result in DNA cleavage, because, if true, such an event would have had predominantly resulted in cleavage at a location somewhere in between the two target sites where the two enzymes converge, which we did not observe. Nicks on dsDNA upon stalling of enzyme due to structural constraints of a DNA -protein complex or due to the presence of a roadblock has been observed in the case of Type I REases (52,53).

Based on the location of the nuclease domain with respect to the target site of a Type ISP REase or MmeI bound to DNA, we predict that in the head-to-head arrangement the nuclease domain of the stationary SauUSI will be engaged with the DNA on the flanking region (Figure 8B). As a result, DNA cleavage by the stationary SauUSI will occur close to the target site on the flanking side resulting in two DNA fragments. The resulting fragment from the flanking side will not have a target site. The other fragment will have two target sites, and will also have the stationary and the stalled SauUSI molecules bound to the cleaved end. Successively, another molecule of SauUSI can bind to the free target site of this fragment and can, upon ATP hydrolysis, catalyze DNA cleavage close to the site at the other flanking region.

A surprising discovery that we made was the shredding by SauUSI of a DNA fragment having target sites at both its ends: a feature never observed before in the case of other REases and endonucleases. While shredding of DNA due to multiple nicks by Type I and Type ISP REases has been reported previously, the shredding was limited to the region around the site of dsDNA break (22,54). In contrast, the shredding by SauUSI was along the entire length of the DNA fragment caused by multiple cuts, which appeared as a smear on the agarose gel. We propose a model for DNA shredding in which a translocating SauUSI starting from a free target site converges and piles-up against the stationary and the stalled SauUSI molecules at another target site. At a higher concentration of the enzyme, more molecules of SauUSI would pile-up. The inefficient nucleolytic activity of the piled-up enzymes can result in random dsDNA break at multiple positions away from the target site, resulting in DNA fragments of varying lengths. The pile-up model, thus, not only explains DNA shredding but also accounts for the observed increase in shredding with increasing enzyme concentration.

We predict a similar mode of cleavage to occur in the case of a head-to-tail arrangement of SauUSI on a two-/multi-site substrate (Supplementary Figure S12). However, in this arrangement, the convergence of the translocating SauUSI with a stationary SauUSI will result in cleavage close to the target site along the central region side. This will result in two DNA fragments each with one target site, with one site bound to the stalled SauUSI and the other to the stationary SauUSI. The DNA with the stalled SauUSI will have a free target site from where more SauUSI molecules can initiate translocation and pile-up against the stalled enzyme, especially at high enzyme concentration. As proposed above, the pile-up can result in multiple dsDNA breaks to cause DNA shredding.

In the tail-to-tail arrangement of SauUSI, the target-site-bound enzymes will never converge (Figure 8A). DNA cleavage in the absence of convergence may be observed because of the enzyme's ability to cleave a single-site substrate. We predict that upon allosteric activation of ATP hydrolysis, the target-site-bound stationary enzyme cuts close to the site resulting in dsDNA break as in the case of a single-site substrate (Figure 8B, Supplementary Figures S13 and S14). DNA shredding will not occur in this case. It is possible that in the case of a single-site substrate or a substrate having target sites in a tail-to-tail orientation where the dsDNA breaks occur close to the target site, the ATPase of SauUSI functions as a switch to activate the nuclease, akin to that observed in the case of Type III REases (23,24). The additional nicks that we observed away from the target site in the case of the single-site substrate may be a result of a transiently stalled translocating enzyme. In the case of palindromic target sites, the reduced efficiency of single-site cleavage in comparison to the two-site cleavage would mean that a convergence-based cleavage will be more rapid rather than a switch-based event (Figure 8B). Therefore, though the probabilities of SauUSI binding in different orientations are the same, the orientations that facilitate convergence would cleave DNA faster than the orientations that would rely on the switch-based mechanism of cleavage.

**Figure 9.**
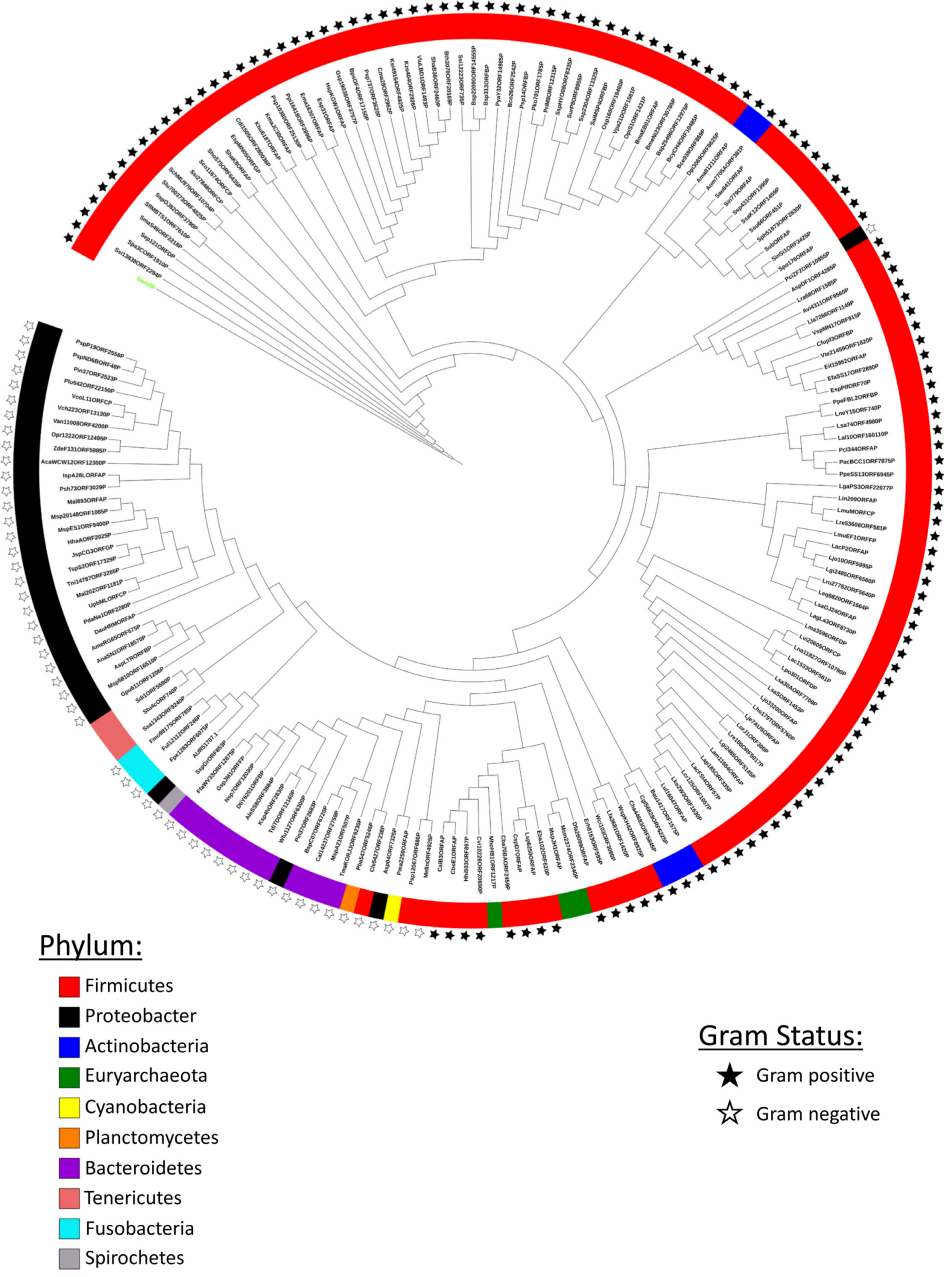
Distribution of SauUSI-like enzymes. A phenogram representing the distribution of SauUSI family of proteins across different bacterial phyla. The color on the strip represents the bacterial phyla, and the star represents if the organism is Gram-positive or Gram-negative. No star is an indication of an archaeal species or species with no cell wall. The node highlighted in fluorescent green is SauUSI. The gene and species name represented in the phenogram are given in Supplementary Table S3.

Shredding by SauUSI should result in extensive and irreparable damage to the integrity of any DNA recognized as foreign by the enzyme, thus making the enzyme a potent barrier against HGT. A chink in this armor is non-methylation or absence of the SauUSI target site, which will prevent the DNA from being recognized as foreign (13,18). SauUSI is not unique to *S. aureus*; a search for homologs of SauUSI revealed its presence in many important lineages of bacteria. A phenogram so generated indicated the occurrence of SauUSI-like enzymes in both Gram-positive and Gram-negative bacteria with a major representation of the enzymes in Firmicutes (Figure 9, Supplementary Table S3). Certain archaea belonging to the phylum Euryarchaeota were also found to have homologs of SauUSI. The presence of the homologs of SauUSI in minimalistic-genome bacteria belonging to the phylum Tenericutes was indicative of the importance of these enzymes as barriers to HGT. The phenogram also suggested to the acquisition of SauUSI-like enzyme by Gram-negative *Proteus cibarius* from Gram-positive Firmicutes. The analysis revealed that SauUSI-like enzymes are common, and may function as important regulators of HGT in bacteria.

## DATA AVAILABILITY

The atomic coordinates and structure factors have been deposited with accession code 7CLG.

## Supplementary Material

gkab042_Supplemental_Files
