# Supplementary material for: Mechanism of DNA cleavage by the endonuclease SauUSI: a major barrier to horizontal gene transfer and antibiotic resistance in Staphylococcus aureus
Source: Nucleic Acids Res. 2021 Feb 3;49(4):2161–78. doi: 10.1093/nar/gkab042 (PMC7913695; doi:10.1093/nar/gkab042)

Plasmid (*pUC 18*) used for the cleavage assay in figure 1a

TCGCGCGTTTCGGTGATGACGGTGAAAACCTCTGACACATGCAGCTCCCGGAGACGGTCACAGCTTGTCTGTAAG
CGGATGCCGGGAGCAGACAAGCCCGTCAGGGCGCGTCAGCGGGTGTTGGCGGGTGTCGGGGCTGGCTTAACTAT
GCGGCATCAGAGCAGATTGTACTGAGAGTGCACCATATGCGGTGTGAAATACCGCACAGATGCGTAAGGAGAAA
ATACCGCATCAGGCGCCATTCGCCATTCAGGCTGCGCAACTGTTGGGAAGGGCGATCGGTGCGGGCCTCTTCGCT
ATTACGCCAGCTGGCGAAAGGGGGATGTGCTGCAAGGCGATTAAGTTGGGTAACGCCAGGGTTTTCCCAGTCAC
GACGTTGTAAAACGACGGCCAGTGCCAAGCTTGCATGCCTGCAGGTCGACTCTAGAGGATCCCCGGGTACCGAGC
TCGAATTCGTAATCATGGTCATAGCTGTTTCCTGTGTGAAATTGTTATCCGCTCACAATTCCACACAACATACGAGC
CGGAAGCATAAAGTGTAAAGCCTGGGGTGCCTAATGAGTGAGCTAACTCACATTAATTGCGTTGCGCTCACTGCC
CGCTTTCCAGTCGGGAAACCTGTCGTGCCAGCTGCATTAATGAATCGGCCAACGCGCGGGGAGAGGCGGTTTGCG
TATTGGGCGCTCTTCCGCTTCCTCGCTCACTGACTCGCTGCGCTCGGTCGTTCGGCTGCGGCGAGCGGTATCAGCT
CACTCAAAGGCGGTAATACGGTTATCCACAGAATCAGGGGATAACGCAGGAAAGAACATGTGAGCAAAAGGCCA
GCAAAAGGCCAGGAACCGTAAAAAGGCCGCGTTGCTGGCGTTTTTCCATAGGCTCCGCCCCCCTGACGAGCATCA
CAAAAATCGACGCTCAAGTCAGAGGTGGCGAAACCCGACAGGACTATAAAGATACCAGGCGTTTCCCCCTGGAAG
CTCCCTCGTGCGCTCTCCTGTTCCGACCCTGCCGCTTACCGGATACCTGTCCGCCTTTCTCCCTTCGGGAAGCGTGG
CGCTTTCTCAAAGCTCACGCTGTAGGTATCTCAGTTCGGTGTAGGTCGTTCGCTCCAAGCTGGGCTGTGTGCACGA
ACCCCCCGTTCAGCCCGACCGCTGCGCCTTATCCGGTAACTATCGTCTTGAGTCCAACCCGGTAAGACACGACTTAT
CGCCACTGGCAGCAGCCACTGGTAACAGGATTAGCAGAGCGAGGTATGTAGGCGGTGCTACAGAGTTCTTGAAG
TGGTGGCCTAACTACGGCTACACTAGAAGAACAGTATTTGGTATCTGCGCTCTGCTGAAGCCAGTTACCTTCGGAA
AAAGAGTTGGTAGCTCTTGATCCGGCAAACAAACCACCGCTGGTAGCGGTGGTTTTTTTGTTTGCAAGCAGCAGAT
TACGCGCAGAAAAAAAGGATCTCAAGAAGATCCTTTGATCTTTTCTACGGGGTCTGACGCTCAGTGGAACGAAAA
CTCACGTTAAGGGATTTTGGTCATGAGATTATCAAAAAGGATCTTCACCTAGATCCTTTTAAATTAAAAATGAAGTT
TTAAATCAATCTAAAGTATATATGAGTAAACTTGGTCTGACAGTTACCAATGCTTAATCAGTGAGGCACCTATCTCA
GCGATCTGTCTATTTCGTTCATCCATAGTTGCCTGACTCCCCGTCGTGTAGATAACTACGATACGGGAGGGCTTACC
ATCTGGCCCCAGTGCTGCAATGATACCGCGAGACCCACGCTCACCGGCTCCAGATTTATCAGCAATAAACCAGCCA
GCCGGAAGGGCCGAGCGCAGAAGTGGTCCTGCAACTTTATCCGCCTCCATCCAGTCTATTAATTGTTGCCGGGAA
GCTAGAGTAAGTAGTTCGCCAGTTAATAGTTTGCGCAACGTTGTTGCCATTGCTACAGGCATCGTGGTGTCACGCT
CGTCGTTTGGTATGGCTTCATTCAGCTCCGGTTCCCAACGATCAAGGCGAGTTACATGATCCCCCATGTTGTGCAAA
AAAGCGGTTAGCTCCTTCGGTCCTCCGATCGTTGTCAGAAGTAAGTTGGCCGCAGTGTTATCACTCATGGTTATGG
CAGCACTGCATAATTCTCTTACTGTCATGCCATCCGTAAGATGCTTTTCTGTGACTGGTGAGTACTCAACCAAGTCA
TTCTGAGAATAGTGTATGCGGCGACCGAGTTGCTCTTGCCCGGCGTCAATACGGGATAATACCGCGCCACATAGC
AGAACTTTAAAAGTGCTCATCATTGGAAAACGTTCTTCGGGGCGAAAACTCTCAAGGATCTTACCGCTGTTGAGAT
CCAGTTCGATGTAACCCACTCGTGCACCCAACTGATCTTCAGCATCTTTTACTTTCACCAGCGTTTCTGGGTGAGCA
AAAACAGGAAGGCAAAATGCCGCAAAAAAGGGAATAAGGGCGACACGGAAATGTTGAATACTCATACTCTTCCTT
TTTCAATATTATTGAAGCATTTATCAGGGTTATTGTCTCATGAGCGGATACATATTTGAATGTATTTAGAAAAATAA
ACAAATAGGGGTTCCGCGCACATTTCCCCGAAAAGTGCCACCTGACGTCTAAGAAACCATTATTATCATGACATTA
ACCTATAAAAATAGGCGTATCACGAGGCCCTTTCGTC

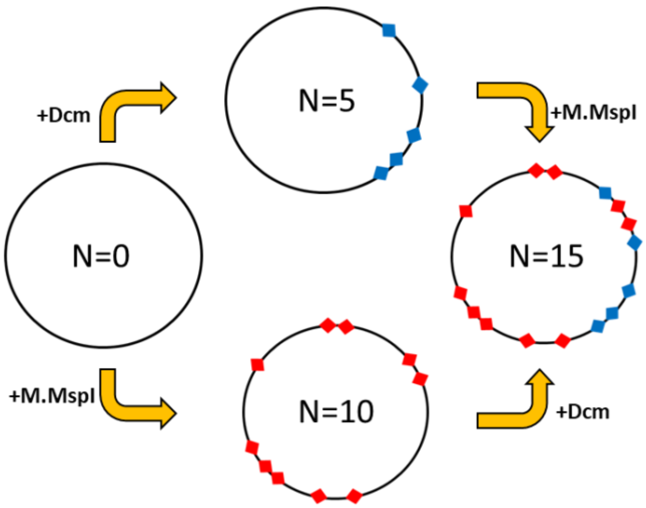
+Dcm
N=5
+M.MspI
N=0
N=15
+M.MspI
N=10
+Dcm

Parent plasmid (*pHis 17* derivative) used to generate DS 1, SS 1, SS 2 and TS.

TGAAGACGAAAGGGCCTCGTGATACGCCTATTTTTATAGGTTAATGTCATGATAATAATGGTTTCTTAGACGTCAG
GTGGCACTTTTCGGGGAAATGTGCGCGGAACCCCTATTTGTTTATTTTTCTAAATACATTCAAATATGTATCCGCTC
ATGAGACAATAACCCTGATAAATGCTTCAATAATATTGAAAAAGGAAGAGTATGAGTATTCAACATTTCCGTGTCG
CCCTTATTCCCTTTTTTGCGGCATTTTGCCTTCCTGTTTTTGCTCACCCAGAAACGCTGGTGAAAGTAAAAGATGCTG
AAGATCAGTTGGGTGCACGAGTGGGTTACATCGAACTGGATCTCAACAGCGGTAAGATCCTTGAGAGTTTTCGCC
CCGAAGAACGTTTTCCAATGATGAGCACTTTTAAAGTTCTGCTATGTGGCGCGGTATTATCCCGTGTTGACGCCGG
GCAAGAGCAACTCGGTCGCCGCATACACTATTCTCAGAATGACTTGGTTGAGTACTCACCAGTCACAGAAAAGCAT
CTTACGGATGGCATGACAGTAAGAGAATTATGCAGTGCTGCCATAACCATGAGTGATAACACTGCGGCCAACTTA
CTTCTGACAACGATCGGAGGACCGAAGGAGCTAACCGCTTTTTTGCACAACATGGGGGATCATGTAACTCGCCTTG
ATCGTTGGGAACCGGAGCTGAATGAAGCCATACCAAACGACGAGCGTGACACCACGATGCCTGCAGCAATGGCA
ACAACGTTGCGCAAACTATTAACTGGCGAACTACTTACTCTAGCTTCCCGGCAACAATTAATAGACTGGATGGAGG
CGGATAAAGTTGCAGGACCACTTCTGCGCTCGGCCCTTCCGGCTGGCTGGTTTATTGCTGATAAATCTGGAGCCGG
TGAGCGTGGGTCTCGCGGTATCATTGCAGCACTGGGGCCAGATGGTAAGCCCTCCCGTATCGTAGTTATCTACACG
ACGGGGAGTCAGGCAACTATGGATGAACGAAATAGACAGATCGCTGAGATAGGTGCCTCACTGATTAAGCATTG
GTAACTGTCAGACCAAGTTTACTCATATATACTTTAGATTGATTTAAAACTTCATTTTTAATTTAAAAGGATCTAGGT
GAAGATCCTTTTTGATAATCTCATGACCAAAATCCCTTAACGTGAGTTTTCGTTCCACTGAGCGTCAGACCCCGTAG
AAAAGATCAAAGGATCTTCTTGAGATCCTTTTTTTCTGCGCGTAATCTGCTGCTTGCAAACAAAAAAACCACCGCTA
CCAGCGGTGGTTTGTTTGCCGGATCAAGAGCTACCAACTCTTTTTCCGAAGGTAACTGGCTTCAGCAGAGCGCAGA
TACCAAATACTGTCCTTCTAGTGTAGCCGTAGTTAGGCCACCACTTCAAGAACTCTGTAGCACCGCCTACATACCTC
GCTCTGCTAATCCTGTTACCAGTGGCTGCTGCCAGTGGCGATAAGTCGTGTCTTACCGGGTTGGACTCAAGACGAT
AGTTACCGGATAAGGCGCAGCGGTCGGGCTGAACGGGGGGTTCGTGCACACAGCCCAGCTTGGAGCGAACGACC
TACACCGAACTGAGATACCTACAGCGTGAGCTATGAGAAAGCGCCACGCTTCCCGAAGGGAGAAAGGCGGACAG
GTATCCGGTAAGCGGCAGGGTCGGAACAGGAGAGCGCACGAGGGAGCTTCCAGGGGGAAACGCCTGGTATCTTT
ATAGTCCTGTCGGGTTTCGCCACCTCTGACTTGAGCGTCGATTTTTGTGATGCTCGTCAGGGGGGCGGAGCCTATG
GAAAAACGCCAGCAACGCGGCCTTTTTACGGTTCCTGGCCTTTTGCTGGCCTTTTGCTCACATGTTCTTTCCTGCGTT
ATCCCCTGATTCTGTGGATAACCGTATTACCGCCTTTGAGTGAGCTGATACCGCTCGCCGCAGCCGAACGACCGAG
CGCAGCGAGTCAGTGAGCGAGGAAGCGGAAGAGCGCCTGATGCGGTATTTTCTCCTTACGCATCTGTGCGGTATT
TCACACCGCAATGGTGCACTCTCAGTACAATCTGCTCTGATGCCGCATAGTTAAGCCAGTATACACTCCGCTATCGC
TACGTGACTGGGTCATGGCTGCGCCCCGACACCCGCCAACACCCGCTGACGCGCCCTGACGGGCTTGTCTGCTCCC
GGCATCCGCTTACAGACAAGCTGTGACCGTCTCCGGGAGCTGCATGTGTCAGAGGTTTTCACCGTCATCACCGAAA
CGCGCGAGGCAGGATCTCGATCCCGCGAAATTAATACGACTCACTATAGGGAGACCACAACGGTTTCCCTCTAGA
AATAATTTTGTTTAACTTTAAGAAGGAGATATACATATGAGCGGTGAGATAAAAAGAATTGATTTAATAAAAAAAA
TGGCGGTCTTTCAGGATTTTAGCTGGTCTTCAGCTGTCAGAGATGAAGGTGATAATGTTGTTGAGTTTAAAAAAAT
TAATATCTTTTATGGCCGCAATTATTCGGGAAAAACTACCCTTTCTCGAATTTTCAGAGCAATGGAAACCGGCTCCA
TTTCAGACAAATATACTTCACCTGAATTCCAGTTGAGTTTTGATGATGAGACTAATGTTACCCAGAAATCTCTGAAT
GGACATGATCAGGTTATTCGGGTTTTCAATGAGGATTTTGTCAAAGAGAATCTTCGCTTTATCGTTGATGATCAGC
ATACTATCAACTCGTTTGCAATTTTGGGTGAAGATAACGCCAAGCTTGAGAAAGAAATCAAACAGCATGAAATCGA
TCTTGGTTCGGAAGAAGACAAATCTGGCCTGATTGGTAAGATGCTTGAAGCTGTTGAGAATCACCGGTTGGCTAC
AAAGAAGCATAGCGAAAGGTCTTCTGGCTTGGAAGGTAAATTAAGAGATAAGGCTAACAAAACCGGAACTGGCA
TTAAGCATAATAAAACATTTGGTGATGCGAACTATAATTTACCAAAGATTAAGGAAGACATAAAAACAGTCACTGC
CAACTCGTATACTCCGATCACTTTTGAGCAAGTTGACAAGTATCATGAACTCTTGAAAGAAGAATCTAAGGAGAAA
ATTCCGGAATCCACAGCCTTCAACCTTAAAAACTCGACTTTCGTCACCCAGGGCAAAAGAGCTGGTAGAAAAAAAG
ATCCAAGCAGCAGATCCGATTCAGGAATTGCTTAATGATGCTGTCCTTGAAGCATGGGTGCGGAATGGACGGGAA

CATCATAAAAACAAAAGAACAAAATGTGCTTTTTGTGGCAGTGATCTTCCGGCAGATCTATGGGAAAAATTGGATA
AGCATTTTAATCAGGAATCTGAAGATTTGCGAATTGCCATTGAAGCTTTGCTTACGTCTATTGATGTCGAAAAGAG
TCGTATACCTAATCTGCTTAAAATCAAGAGCTCGGATTTTTATTCGAATTTCAGTTCAGATCTGGAAACACTGGGAA
AACAATTTTCTGCCCAATCAACTGCGTATTGCAAAACACTTGAATCGATAAAAGAACAACTTGAGAAACGCAAAAG
TGATATTTTCACTCCTCTTATCTTTGATGATCCGACATCCGTTGAGCAGGAGTTGAATGCTGTTCGAGACTTTTATG
AAGAGCTGAGGAATGAATCAAACCAATTTAGAGCTTCATTAAGTGCTCAGCAATCAGAGGCCCGCGCAGCTCTTC
GCCTGCATGAGGTTTTCACATTTATAACAGACATAAAATATGCTGATGAATGCAAAGCTATTGAAGCATTAAAAAA
GGCCATGGATAAGGCGGAGAAAGCCAAGAGCACCGCAAAGGCAGAAGTTGACACCAAGAAAGCTAAAATCAGC
GAACTAAAAGCCCAGTTGAAAGATGAAAGCAAGGGTGCCGATAGGGTTAATGATTATCTGAATAATTATTTCGGG
CACCCGTTTTTATCTCTTAAAGCAATAGAGGATACATCCGATGATACTTCCACTGGATACCGATTTGAAGTAACCAG
AAATAACAAAAAAGCTTTTCATCTCAGCGAAGGAGAATGTGGGCTGATTGCTTTCTGCTATTTTATGGCCAAGTTG
GATGATGTTGAAACGAAGGGAAGCCAACCAATAATATGGATCGATGATCCAATCTCCAGCCTAGATGCAAATCAT
ATCTTTTTTGTCTACAGCCTGATTAATGCCGAGATTGTTAAGCCCAAGAACTATAAACAACTTTTCATTTCTACTCAT
AATCTAGATTTCTTGAAGTATTTAAAAAGAATTTCTAATGACTATAAAGGAAAAGGTGAAAACAAGGTAAAGATAC
GGGAGTTTTTTCTAATTGAACGCAACGGTAATTCCAGTTGTCTGTCGTTAATGCCATGCTATTTAAAGAGATATGTG
ACTGAATTTAATTACCTTTTTCACCAAATCTATAAGAGTGCAACAACCGAATCAATAAATGACAAGAACTACAAAG
ATTTCTATAGCTTTGGGAATAATGCCCGAAAATTCTTTGAAATTTATTTGTATTACAAATACCCAAATCAGGGAATG
AACGCAGAAACTCTTAGTTATTTCTTTGGAGATGAAAAAGTTCCAGCTATTTTGACTGATAGGATCAATAACGAAT
ATTCTCATTTGGCTGGTGTTTTTGAAAGAGGTTCAACACCTATTGAAGTTCCTGAAATGACGACAACGGCAAACTTT
ATTCTTCAGAAGATTAGAGAGAAAGACCTAGATCAGTATTCGGCGTTACTGAAAAGTATTGGTGTAAAAGATGAG
GAACCCCAAACAGCAGTGGAGGGTCAAGGATCCCATCATCATCATCATCATTAAAAGCTTCACCACCACCACCACC
ACTAAGAATTCCATCATCATCATCATCATTAACGATCCGGCTGCTAACAAAGCCCGAAAGGAAGCTAGGTTGGCTG
CTGCCACCGCTGAGCAATAACTAGCATAACCCCTTGGGGCCTCTAAACGGGTCTTGAGGGGTTTTTTGCTGAAAGG
AGGAACTATATCCGGATAATTCT

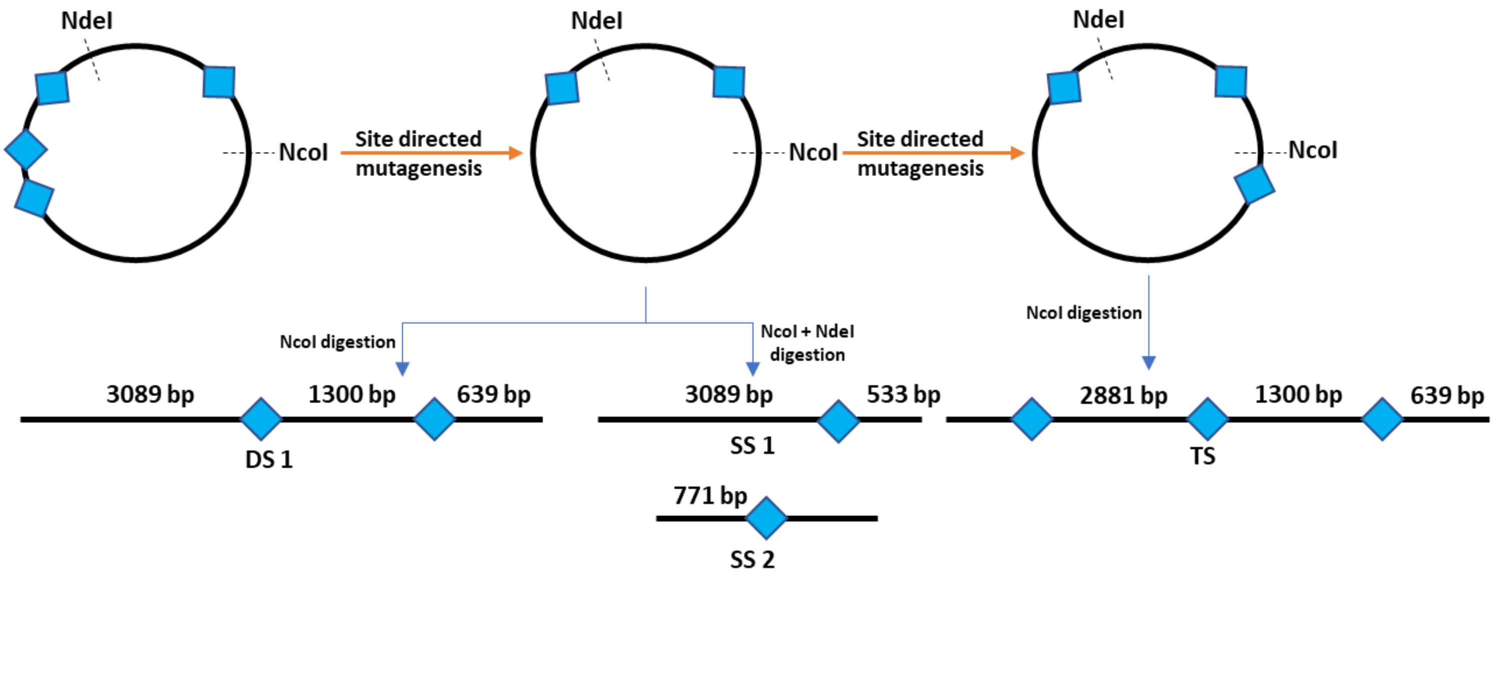

Parent plasmid (*pHis 17* derivative) used to generate DS 2.

TGAAGACGAAAGGGCCTCGTGATACGCCTATTTTTATAGGTTAATGTCATGATAATAATGGTTTCTTAGACGTCAG
GTGGCACTTTTCGGGGAAATGTGCGCGGAACCCCTATTTGTTTATTTTTCTAAATACATTCAAATATGTATCCGCTC
ATGAGACAATAACCCTGATAAATGCTTCAATAATATTGAAAAAGGAAGAGTATGAGTATTCAACATTTCCGTGTCG
CCCTTATTCCCTTTTTTGCGGCATTTTGCCTTCCTGTTTTTGCTCACCCAGAAACGCTGGTGAAAGTAAAAGATGCTG
AAGATCAGTTGGGTGCACGAGTGGGTTACATCGAACTGGATCTCAACAGCGGTAAGATCCTTGAGAGTTTTCGCC
CCGAAGAACGTTTTCCAATGATGAGCACTTTTAAAGTTCTGCTATGTGGCGCGGTATTATCCCGTGTTGACGCCGG
GCAAGAGCAACTCGGTCGCCGCATACACTATTCTCAGAATGACTTGGTTGAGTACTCACCAGTCACAGAAAAGCAT
CTTACGGATGGCATGACAGTAAGAGAATTATGCAGTGCTGCCATAACCATGAGTGATAACACTGCGGCCAACTTA
CTTCTGACAACGATCGGAGGACCGAAGGAGCTAACCGCTTTTTTGCACAACATGGGGGATCATGTAACTCGCCTTG
ATCGTTGGGAACCGGAGCTGAATGAAGCCATACCAAACGACGAGCGTGACACCACGATGCCTGCAGCAATGGCA
ACAACGTTGCGCAAACTATTAACTGGCGAACTACTTACTCTAGCTTCCCGGCAACAATTAATAGACTGGATGGAGG
CGGATAAAGTTGCAGGACCACTTCTGCGCTCGGCCCTTCCGGCTGGCTGGTTTATTGCTGATAAATCTGGAGCCGG
TGAGCGTGGGTCTCGCGGTATCATTGCAGCACTGGGGCCAGATGGTAAGCCCTCCCGTATCGTAGTTATCTACACG
ACGGGGAGTCAGGCAACTATGGATGAACGAAATAGACAGATCGCTGAGATAGGTGCCTCACTGATTAAGCATTG
GTAACTGTCAGACCAAGTTTACTCATATATACTTTAGATTGATTTAAAACTTCATTTTTAATTTAAAAGGATCTAGGT
GAAGATCCTTTTTGATAATCTCATGACCAAAATCCCTTAACGTGAGTTTTCGTTCCACTGAGCGTCAGACCCCGTAG
AAAAGATCAAAGGATCTTCTTGAGATCCTTTTTTTCTGCGCGTAATCTGCTGCTTGCAAACAAAAAAACCACCGCTA
CCAGCGGTGGTTTGTTTGCCGGATCAAGAGCTACCAACTCTTTTTCCGAAGGTAACTGGCTTCAGCAGAGCGCAGA
TACCAAATACTGTCCTTCTAGTGTAGCCGTAGTTAGGCCACCACTTCAAGAACTCTGTAGCACCGCCTACATACCTC
GCTCTGCTAATCCTGTTACCAGTGGCTGCTGCCAGTGGCGATAAGTCGTGTCTTACCGGGTTGGACTCAAGACGAT
AGTTACCGGATAAGGCGCAGCGGTCGGGCTGAACGGGGGGTTCGTGCACACAGCCCAGCTTGGAGCGAACGACC
TACACCGAACTGAGATACCTACAGCGTGAGCTATGAGAAAGCGCCACGCTTCCCGAAGGGAGAAAGGCGGACAG
GTATCCGGTAAGCGGCAGGGTCGGAACAGGAGAGCGCACGAGGGAGCTTCCAGGGGGAAACGCCTGGTATCTTT
ATAGTCCTGTCGGGTTTCGCCACCTCTGACTTGAGCGTCGATTTTTGTGATGCTCGTCAGGGGGGCGGAGCCTATG
GAAAAACGCCAGCAACGCGGCCTTTTTACGGTTCCTGGCCTTTTGCTGGCCTTTTGCTCACATGTTCTTTCCTGCGTT
ATCCCCTGATTCTGTGGATAACCGTATTACCGCCTTTGAGTGAGCTGATACCGCTCGCCGCAGCCGAACGACCGAG
CGCAGCGAGTCAGTGAGCGAGGAAGCGGAAGAGCGCCTGATGCGGTATTTTCTCCTTACGCATCTGTGCGGTATT
TCACACCGCAATGGTGCACTCTCAGTACAATCTGCTCTGATGCCGCATAGTTAAGCCAGTATACACTCCGCTATCGC
TACGTGACTGGGTCATGGCTGCGCCCCGACACCCGCCAACACCCGCTGACGCGCCCTGACGGGCTTGTCTGCTCCC
GGCATCCGCTTACAGACAAGCTGTGACCGTCTCCGGGAGCTGCATGTGTCAGAGGTTTTCACCGTCATCACCGAAA
CGCGCGAGGCAGGATCTCGATCCCGCGAAATTAATACGACTCACTATAGGGAGACCACAACGGTTTCCCTCTAGA
AATAATTTTGTTTAACTTTAAGAAGGAGATATACATATGTCCCATGAATGGCTCATAAGCGCATCGGAAACAATGA
GAAGCATTGGTAATGGGGAAGGTTTGCGGGATAAAGGTGCTGTCGTTGCCAACAATGACGGAGAATTTAACGAA
GGTGATACAAATCGTGAAGAGGATAGCTCTACCATTTTCTCCTTCGATTTTGACGAG GAGATTGTAATGTGTATTG
ACTTTTCTGGAGGAAAACTCGGGTGTTCTATTTTAGATTATCATACCAAGACTTTGAAAGCGTTTGACCAGGACTAC
GTGGTCAATAAAACAACAATTTCGTCTCATGATCTTATCGATGATGCAGATATGTCTTCAAACGACATAAGTCTGTT
ACTTGGACTTTTAATAATGGAAGCAAACCCAACTGTATGCCTAGTGCCGGCAAGATTGGAGGATTGGATTTTCGAC
TACATTAAGACAAAATGCGATGAGATAAACTGTAGGCTAGAACTACAACCAATAAAACGTTTCAAAAAATGGGAC
TTATTGCAGTCATTGCAGTTAAGGGGTCATGATAATCAGACTATATTGAACGACATATTATCAAATAGCAAATTTAC
CACCACAGTCACCTTAGGTACAGTGGGATGCATCCTTGCAAATCATGAACAACTTGGTGAATACAATGACAGTACT
GCTTCAAGTAATATGGTAACAGGACGATTAGTTCAGAATGCTTTTGAAGATGTGATACATGGCATAAGGTATATCG
ACATAAGGGATCGGATGGTACTGGATGAAAATACTATATCGGCTTTGCATATTTTTCCAACAGCTCATAAACTGGG
CCATGATAAGATGATGAGAAATGGTTTCTTTAGTGTATTTGAACTATTCAATCAAGTGTCTTCGGACTACGCCAGG

AGAATTTTGAAGTCTTGGCTTATTAACCCATTAACCAATAAAAAACGGATAGAAACAAGATACAGCATCATAAGAA
CCTTATTGGATAAACAAAACGCCATCATTTTTAGTGACCTTAGCCAATCAATAAAAAGATGTCCAGACGCTTTTGGG
TTTATAAATCAGTTAAGAAGTGGTAAATCGACATTAGGGACATGGTCCAAGGTTGCAAGTTTTTTAGAAAAAGGA
ATAGCTATATTTCAACTGGTGTCGTCCTTGAAATTAGGTTCAGACGAAGCCAACATTTTACATGATATCAAAAATAA
GGTTGATATATCAGCTTTAAAGGAATGTTTGAGAAAAGTAGAAACGGTAATAGATTTTGACACATCAAGAGATAC
CAAGACGCTCACGATAAATACGGGGGTTGACAACAGATTAGATGAATGCAGAAATATTTATAATCATTTGGAAGG
GATCTTACTGGATGTCGCAAGAGAAACTCGAATTTTTTTACTGAATACTATGCCTCAAGAAGATTGTAAGACAACA
AAGAGTTTAGAAAAGTTAGTGAATGCTGTTTATATTCCCCAATTAGGATACTTAGTAACCATTAGCGTCTTAATGGA
ACCTTTATTTGATGGCATTCCAAACCTTCAATGGGAAGAGATCTTTAGGAGTTCAGAAAATATATACTTCAAAAATG
GCAGGGTTTTTGAGCTGGATGAAACATACGGCGATATTTATGGCGCGATTTCAGATTTTGAAATTGAAATCCTGTT
TTCCTTACAAGAACAAATTCTGAGAAGAAAGACTCAACTCACTGCTTACAATATACTGCTTAGTGAACTGGAAATAT
TATTATCGTTCGCTCAAGTGTCTGCTGAAAGGAATTATGCAGAGCCTCAATTGGTGGAAGATGAGTGCATATTGGA
AATTATTAACGGAAGACATGCTTTGTATGAAACATTCCTTGATAATTATATCCCCAATAGCACAATGATTGATGGCG
GGCTATTTTCTGAATTAAGTTGGTGTGAACAAAATAAAGGAAGAATCATTGTAGTCACTGGCGCTAATGCATCTGG
AAAGTCTGTATATCTTACACAGAATGGTTTAATTGTGTACTTGGCACAAATTGGTTGTTTTGTTCCAGCAGAGAGA
GCGAGAATTGGAATAGCGGATAAAATATTAACTAGAATCAGGACTCAAGAAACTGTTTATAAGACTCAAAGTTCCT
TTTTGCTAGATTCTCAACAAATGGCAAAATCACTGAGTTTGGCCACTGAAAAAAGTCTCATTTTAATTGATGAATAC
GGCAAAGGTACTGATATTTTAGATGGACCTTCACTATTTGGTTCCATAATGCTCAATATGTCCAAGAGTGAAAAAT
GTCCACGCATAATCGCATGTACACACTTTCATGAACTATTTAACGAAAATGTACTCACAGAGAATATAAAAGGTAT
CAAACATTACTGCACTGATATACTTATCAGCCAAAAATATAATCTTTTAGAAACAGCACATGTCGGAGAAGATCAT
GAAAGTGAGGGAATCACATTCCTATTCAAAGTAAAGGAGGGGATCTCAAAGCAGTCATTTGGCATACATTGCGCA
AAAGTATGTGGTTTGAGCAGAGATATTGTGGAAAGGGCTGAAGAACTATCTCGCATGATTAATAGAGGCGATGAC
GTAGTTCAGCAATGTGGAAATCTGACCGAAAAGGAGATGAGAGAATTCCAAAAGAATCAGGAAATAGTGAAAAA
GTTTTTATCTTGGGATTTGGATCTCGAAACTACAACAACTTCCGAGAATCTCAGGCTTAAATTGAAAAATTTCCTTC
GCGGATCCCATCATCATCATCATCATTAAAAGCTTCACCACCACCACCACCACTAAGAATTCCATCATCATCATCATC
ATTAACGATCCGGCTGCTAACAAAGCCCGAAAGGAAGCTAGGTTGGCTGCTGCCACCGCTGAGCAATAACTAGCA
TAACCCCTTGGGGCCTCTAAACGGGTCTTGAGGGGTTTTTTGCTGAAAGGAGGAACTATATCCGGATAATTCT

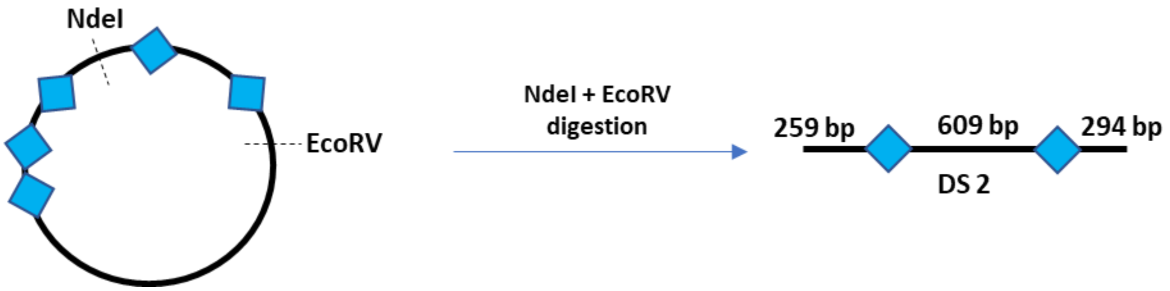

Supplement: gkab042_Supplemental_Files [file gkab042_supplemental_files.zip › Plasmid details.pdf]
