# Supplementary material for: Mechanism of DNA cleavage by the endonuclease SauUSI: a major barrier to horizontal gene transfer and antibiotic resistance in Staphylococcus aureus
Source: Nucleic Acids Res. 2021 Feb 3;49(4):2161–78. doi: 10.1093/nar/gkab042 (PMC7913695; doi:10.1093/nar/gkab042)
Supplement: gkab042_Supplemental_Files [file gkab042_supplemental_files.zip › Supplementary information-rv2.pdf]

Supplementary Information

Mechanism of DNA cleavage by the endonuclease SauUSI – a major barrier to horizontal gene transfer and antibiotic resistance in *Staphylococcus aureus*

Vinayak Sadasivam Tumuluri[1], Vrunda Rajgor[1], Shuang-yong Xu[2], Om Prakash Chauhan[1], Kayarat Saikrishnan[1*]

[1]Department of Biology, Indian Institute of Science Education and Research, Pune, 411008, India.

[2]New England Biolabs Inc., Research Department, Ipswich, MA 01938, USA

[*]Correspondence and requests for materials should be addressed to K.S. (email: saikrishnan@iiserpune.ac.in)

This PDF file includes:

Supplementary Figures S1 to S14
Supplementary Tables S1 to S3

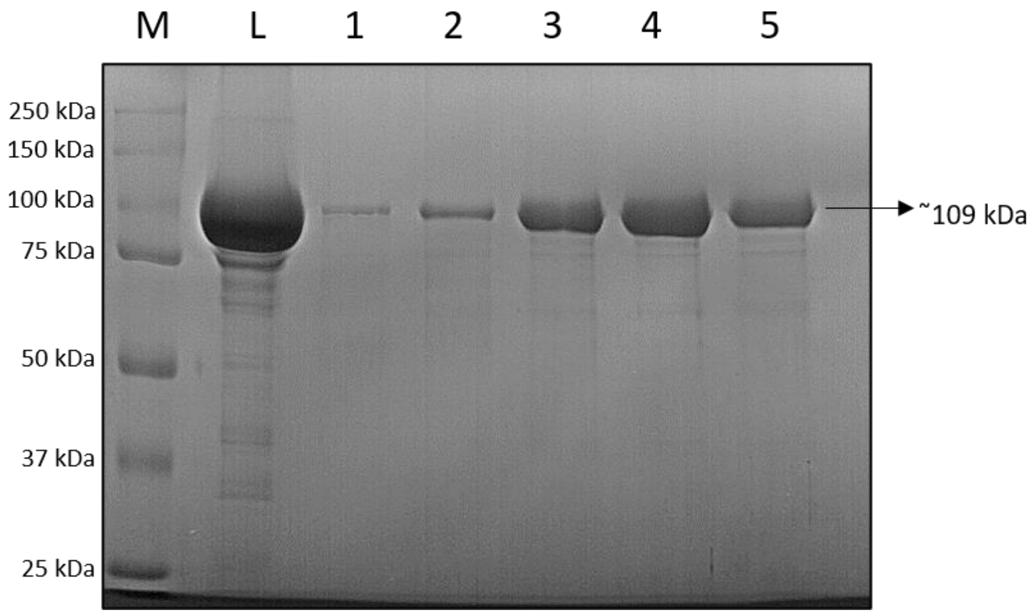

Supplementary figure S1. Representative 10% SDS-PAGE gel for the purification of SauUSI after Superdex 200 size exclusion column. M represents the protein marker, and L represents the sample loaded from the previous column (MonoQ).

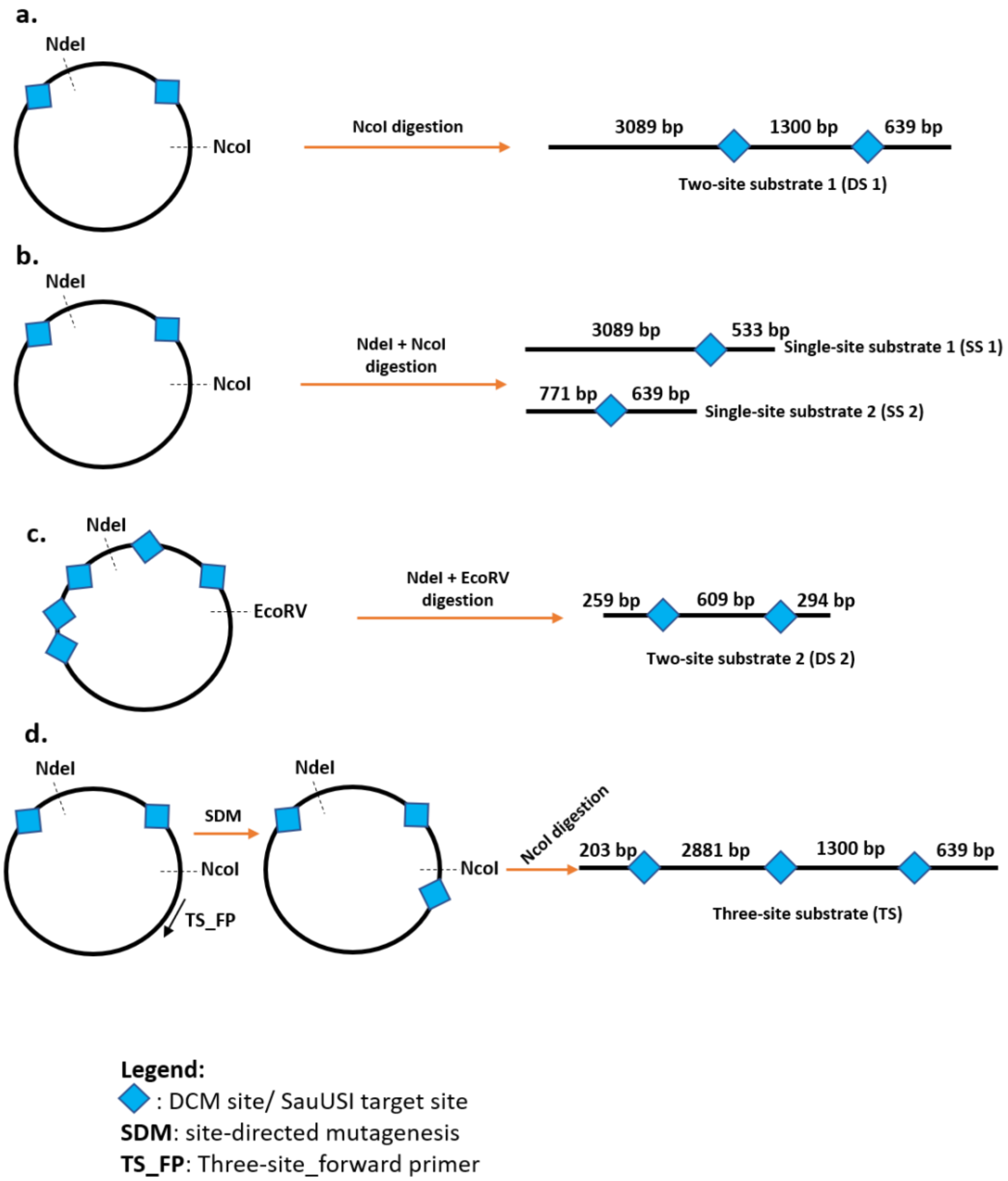

Supplementary figure S2. (a) Schematic of the two-site plasmid (DS 1). The plasmid was grown in a *dcm*$^{+}$ strain of *E. coli* and was purified and linearized with NcoI-HF. (b) Schematic of the single-site substrates SS 1 and SS 2. The two-site plasmid was digested with NcoI-HF and NdeI, and the two fragments were extracted from an agarose gel and purified. (c) DS2 was generated by digesting a plasmid with five sites (grown in a *dcm*$^{+}$ strain of *E.coli*) with NdeI and EcoRV followed by an agarose gel purification (d) The three-site plasmid (TS) was generated using site-directed mutagenesis of the two-site plasmid. The three-site plasmid was processed similar to the two-site plasmid.

a.

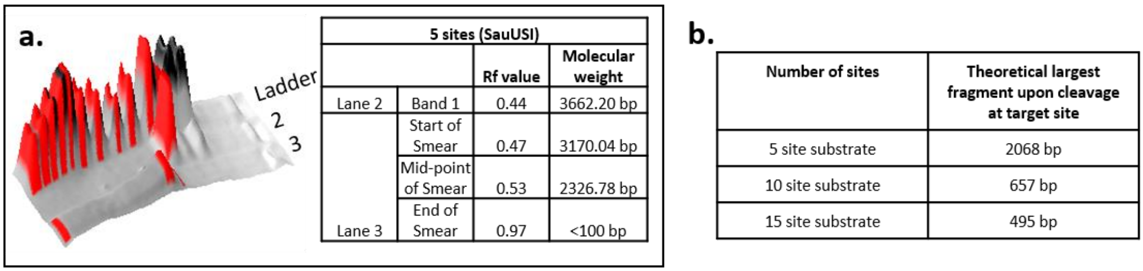

5 sites (SauUSI)			
		Rf value	Molecular weight
Lane 2	Band 1	0.44	3662.20 bp
	Start of Smear	0.47	3170.04 bp
	Mid-point of Smear	0.53	2326.78 bp
Lane 3	End of Smear	0.97	<100 bp

b.

Number of sites	Theoretical largest fragment upon cleavage at target site
5 site substrate	2068 bp
10 site substrate	657 bp
15 site substrate	495 bp

c.

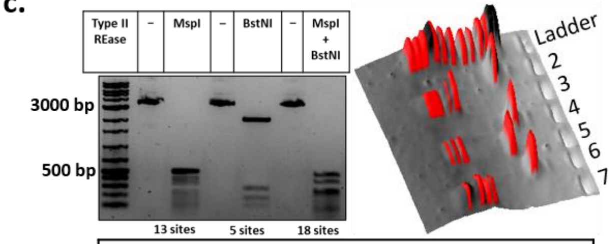

13 sites (MspI)			
		Rf value	Molecular weight
Lane 2	Band 1	0.3	3739.58 bp
	Band 1	0.58	508.31 bp
	Band 2	0.62	391.35 bp
Lane 3	Band 3	0.68	245.10 bp
**5 sites (BstNI)**			
		Rf value	Molecular weight
Lane 4	Band1	0.3	3638.04 bp
	Band 1	0.37	2216.61 bp
	Band 2	0.65	309.71 bp
	Band 3	0.69	238.44 bp
Lane 5	Band 4	0.72	183.58 bp
**18 sites (MspI+BstNI)**			
		Rf value	Molecular weight
Lane 6	Band 1	0.3	3638.04 bp
	Band 1	0.59	474.51 bp
	Band 2	0.62	375.52 bp
	Band 3	0.67	273.63 bp
Lane 7	Band 4	0.74	166.72 bp

d.

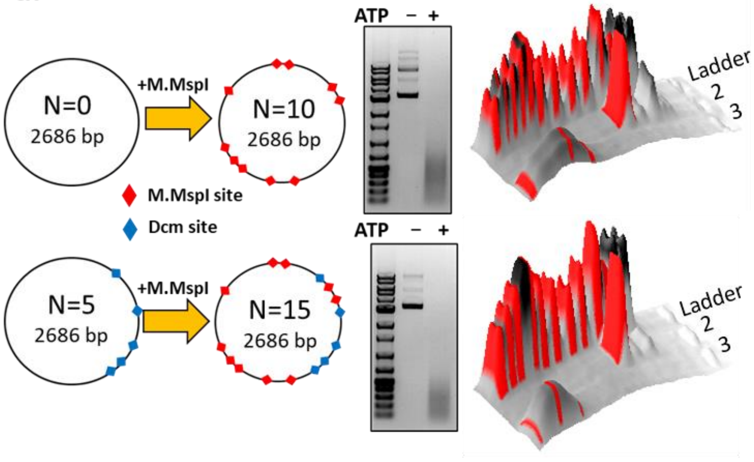

10 sites (SauUSI)			
		Rf value	Molecular weight
Lane 2	Band 1	0.43	3695.96 bp
	Start of Smear	0.7	888.86 bp
	Mid-point of Smear	0.8	532.37 bp
Lane 3	End of Smear	0.97	<100 bp

15 sites (SauUSI)			
		Rf value	Molecular weight
Lane 2	Band 1	0.43	3686.48 bp
	Start of Smear	0.76	621.56 bp
	Mid-point of Smear	0.87	350.60 bp
Lane 3	End of Smear	0.97	<100 bp

Supplementary figure S3. (a) 3-D representation of cleavage of SauUSI on a plasmid with five sites (derived from figure 1a). The red color represents the region of the peak quantified. The adjoining table provides details of the Rf value and the calculated molecular weight. (b) A table containing theoretically largest fragments formed if plasmids were cleaved at the SauUSI target sites. (c) 1% Agarose gel of the cleavage of plasmid having multiple sites (part of the gel also shown in figure 1a) by MspI, BstNI and a combination of the two. 3-D representation of the cleavage pattern. The table of Rf values and calculated molecular weights is also given. (d) Schematic of the plasmid used for cleavage. N represents the number of target sites of SauUSI in a particular plasmid. The names on the arrows are the methyltransferases used

(either *in vitro* or *in vivo*) to generate the methylated target sites of SauUSI. The corresponding cleavage pattern (on a 1% agarose gel) of SauUSI on the respective plasmids. The reactions with and without nucleotide (ATP) are compared against a DNA marker. 3-D representation of cleavage of SauUSI on a plasmid with 10 sites and 15 sites respectively. The red color represents the region of the peak quantified. Since SauUSI cleavage produces smears, therefore the start and mid-points of the peaks are quantified. The adjoining tables provide details of the Rf value and the respective calculated molecular weights.

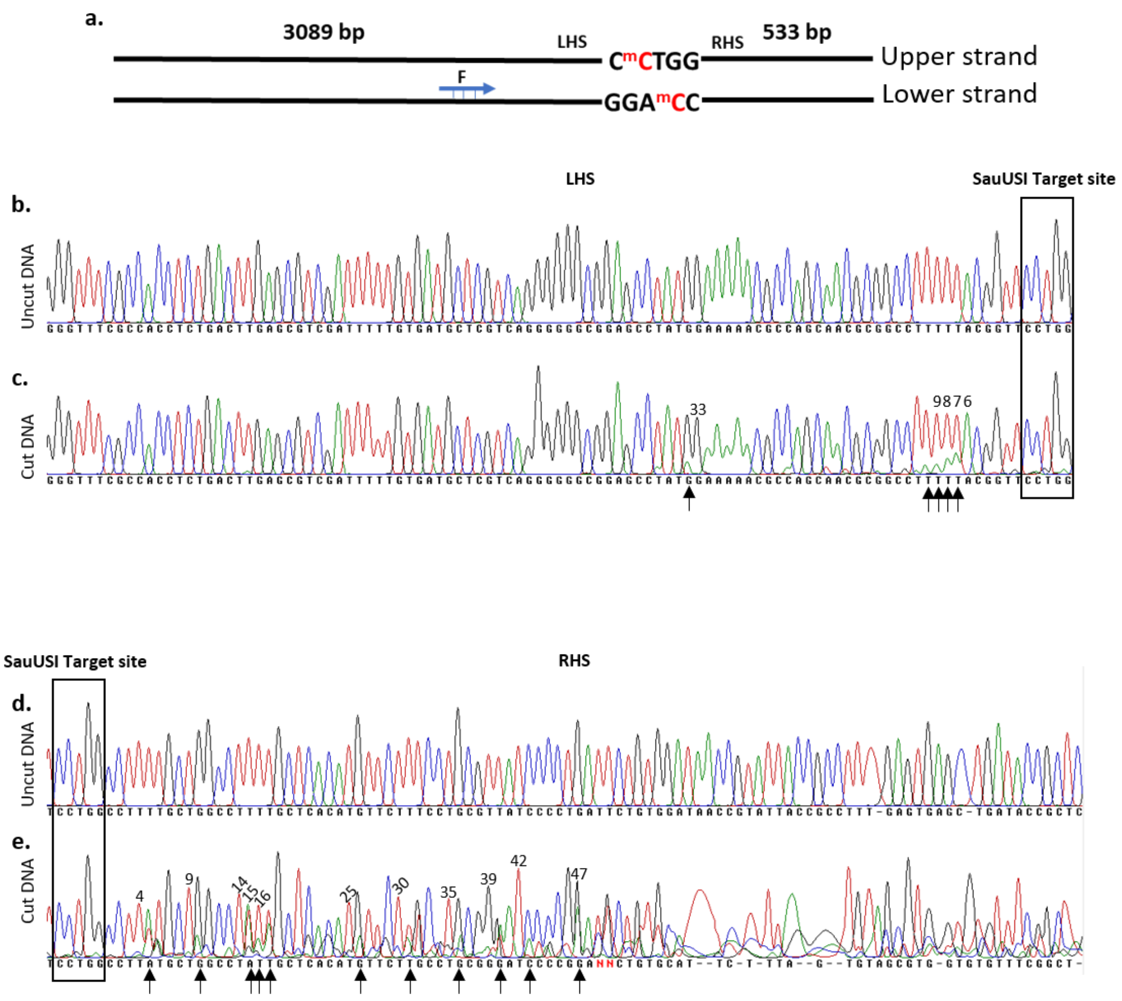

Supplementary figure S4. Sequencing assay (forward direction) to locate nicks in the lower strand: (a) Schematic of the DNA substrate used for sequencing. The cytosines highlighted in red represents methylation. F refers to the forward primer. The DNA preceding the target site is annotated LHS, and the DNA succeeding the target site is annotated RHS. (b) Sequencing of the uncut DNA substrate on the LHS used for the assay. (c) Sequencing of the cleaved fragment of DNA in the forward direction on the LHS. The arrows on the doublet peaks indicate nicks identified by sequencing. The numbers represent the position of cleavage in base-pairs away from the SauUSI target site. (d) Sequencing of the uncut DNA substrate on the RHS used for the assay. (e) Sequencing of the cleaved fragment in the forward direction on the RHS. The arrows on doublet peaks indicate nicks. The numbers represent the position of cleavage in base-pairs away from the SauUSI target site. It is to be noted that the sequencing result gets very noisy (multiple overlapping peaks) >45 bp from the SauUSI target site.

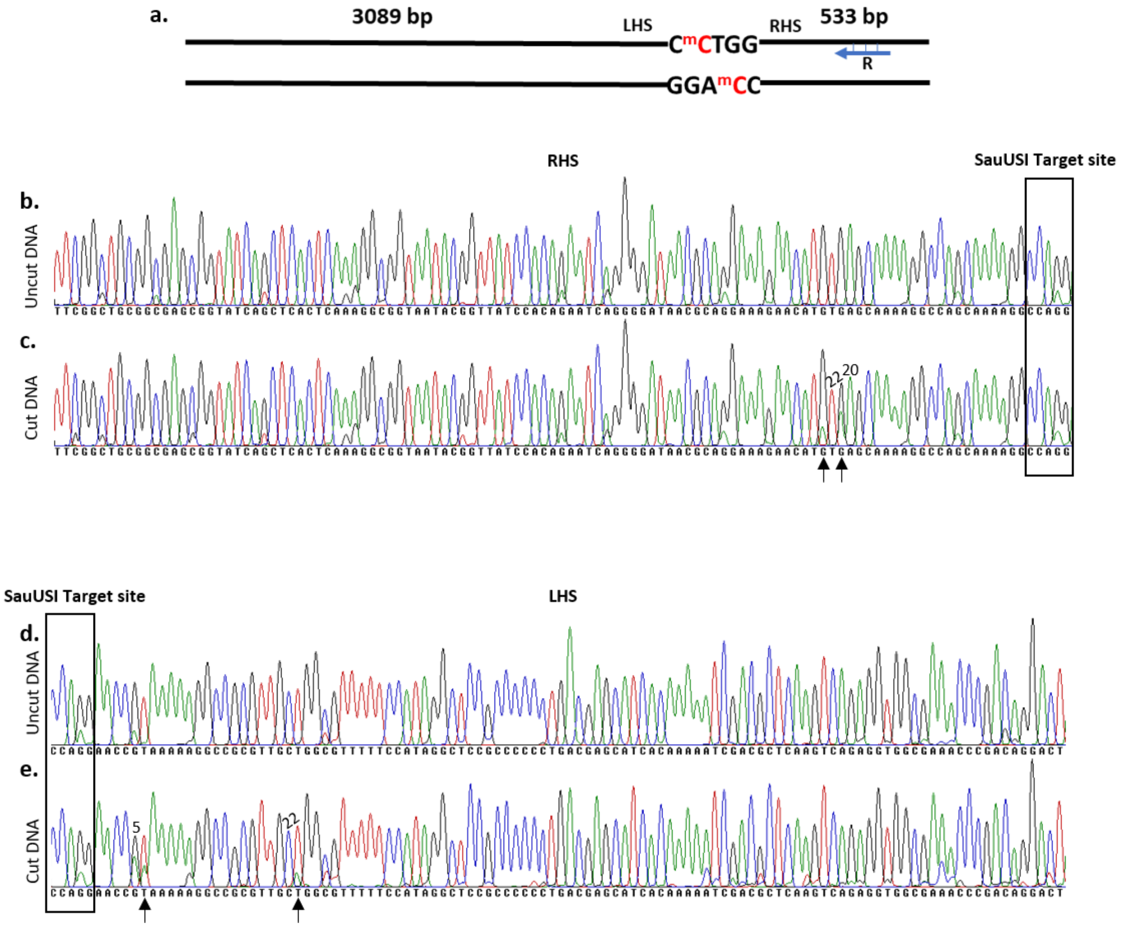

Supplementary figure S5. Sequencing assay (reverse direction) to locate nicks in the upper strand: (a) Schematic of the DNA substrate used for sequencing. The cytosines highlighted in red represents methylation. R refers to the reverse primer. The annotations of LHS and RHS are as in supplementary figure 4 for the sake of convenience. (b) Sequencing of the uncut DNA substrate on the RHS used for the assay. (c) Sequencing of the cleaved fragment of DNA in the reverse direction on the RHS. The arrows on the doublet peaks indicate nicks identified by sequencing. The numbers represent the position of cleavage in base-pairs away from the SauUSI target site. (d) Sequencing of the uncut DNA substrate on the LHS used for the assay. (e) Sequencing of the cleaved fragment in the reverse direction on the LHS. The arrows on the doublet peaks indicate nicks. The numbers represent the position of cleavage in base pairs away from the SauUSI target site.

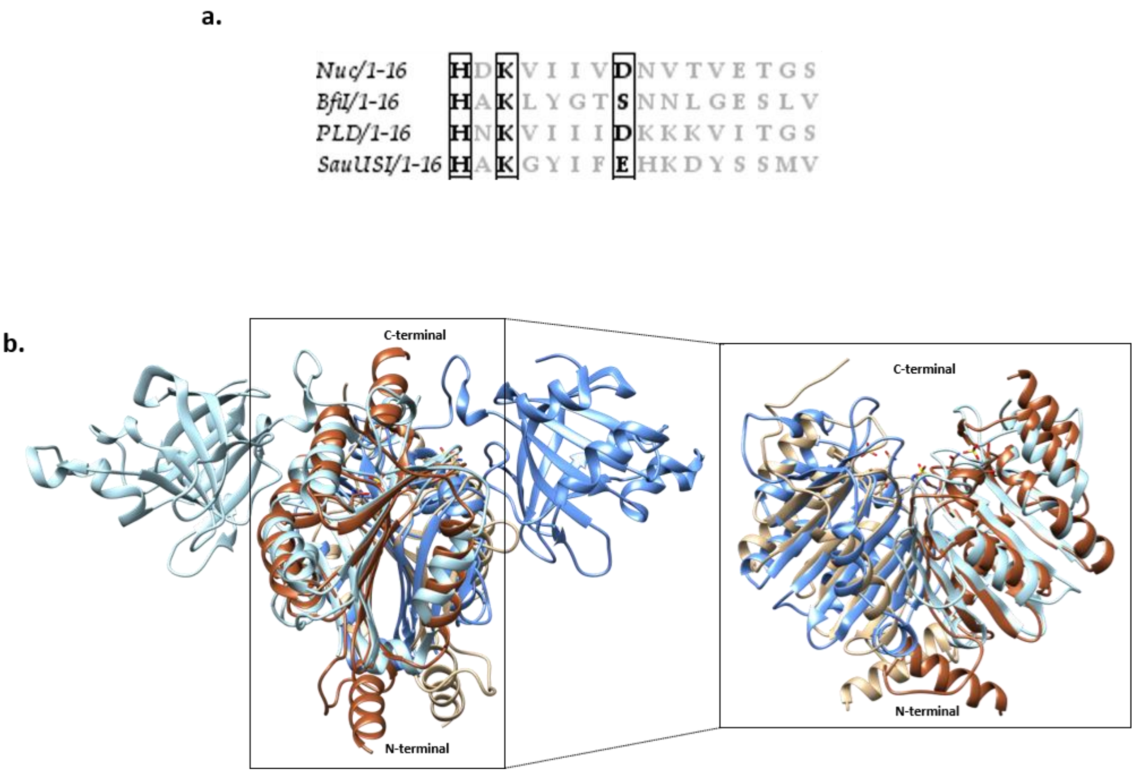

Supplementary figure S6. (a) Multiple sequence alignment of the Phospholipase D (PLD) domain of SauUSI with other members of the PLD family comprising of exonucleases, endonucleases and lipases in humans. The histidine plays the role of the nucleophile causing the break in the phosphodiester bond (b) Superposition of the dimeric nuclease domains (tan and brown) of SauUSI on to the dimeric REase BfiI (light and dark blue) (PDB ID: 2C1L). In the inset is the superposition with the target recognition domain of BfiI removed for clarity, and the molecules are rotated by 90°.

a.

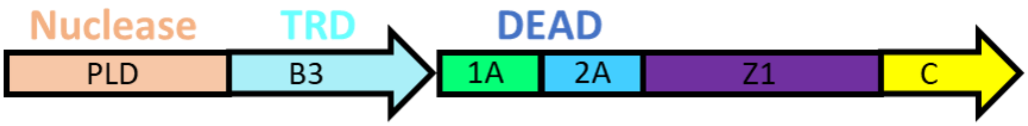

b.

Motif	H(X)K(X)4D/E
Protein	
R.CgII	HGKIYLFH
R.NgoAVII	HGKMYSFK
SauUSI	HAKGYIFE

Proposed motifs based on sequence alignments (R.CgII, R.NgoAVII)

Proposed motifs based on sequence and structural alignments (SauUSI)

Supplementary figure S7. (a) The primary domain architecture of the restriction enzymes CglI and NgoAVII. Both the REases are made of two subunits, a R-subunit and H-subunit. The H-subunit harbors the DEAD-box helicase domain and the accessory domains Z1 and C. On the other hand, the R-subunit harbors the PLD nuclease domain and the B3 target recognition domain. (b) The PLD nuclease active site residues of SauUSI compared with that of R.CglI and R.NgoAVII.

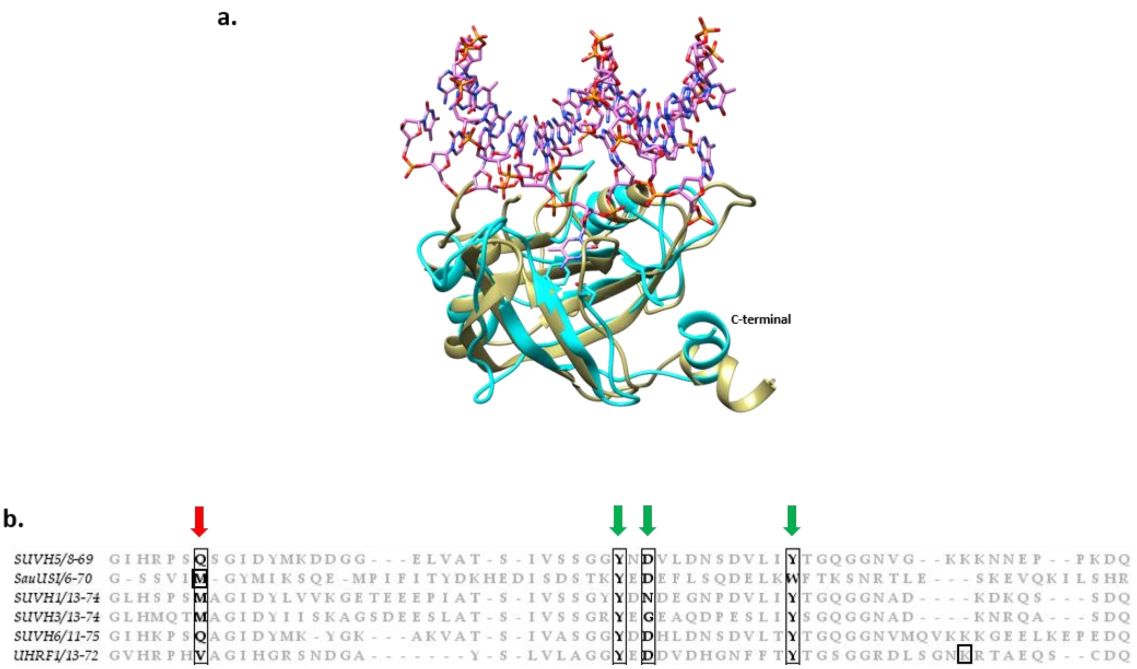

Supplementary figure S8. (a) Structural superposition of the SRA domain of SauUSI on to the DNA-bound SRA domain of SUVH6, a H3K9 histone methyltransferase from *Arabidopsis thaliana* (PDB ID: 6A5N). (b) Multiple sequence alignment of the SRA domain with members of SuvH5 family proteins and UHRF1. The red arrow represents residues that insert into the cavity in the DNA duplex formed by base flipping; Q392 in SuvH5 aligns sequentially and structurally with M829 of SauUSI. The Green arrows represent residues that stabilize the flipped out 5mC either through hydrogen bonding or stacking interactions; D858 interacts through hydrogen bons with the flipped-out base whereas, Y856 and W868 seem to stabilize the 5mC through stacking interactions. However, UHRF1 uses the lysine residue (marked in a box) from the 'NKR' finger to protrude into the DNA helix. The 'NKR' finger doesn't seem to exist in SauUSI.

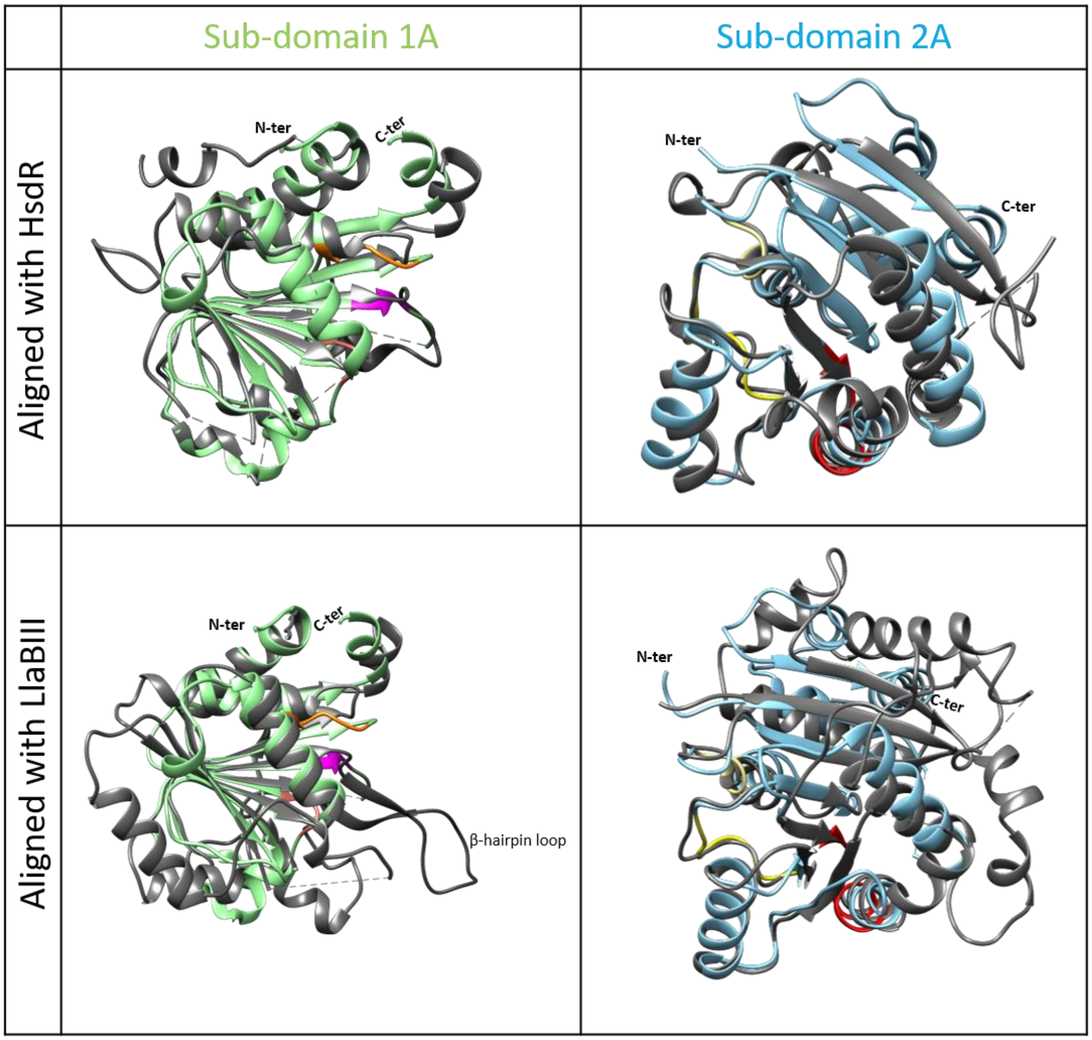

Supplementary figure S9. The 1A and 2A sub-domains of the SF2 helicase-like ATPase domain of SauUSI structurally aligned with the respective subdomains of HsdR (PDB ID: 3H1T) and LlaBIII (PDB ID: 4XQK).

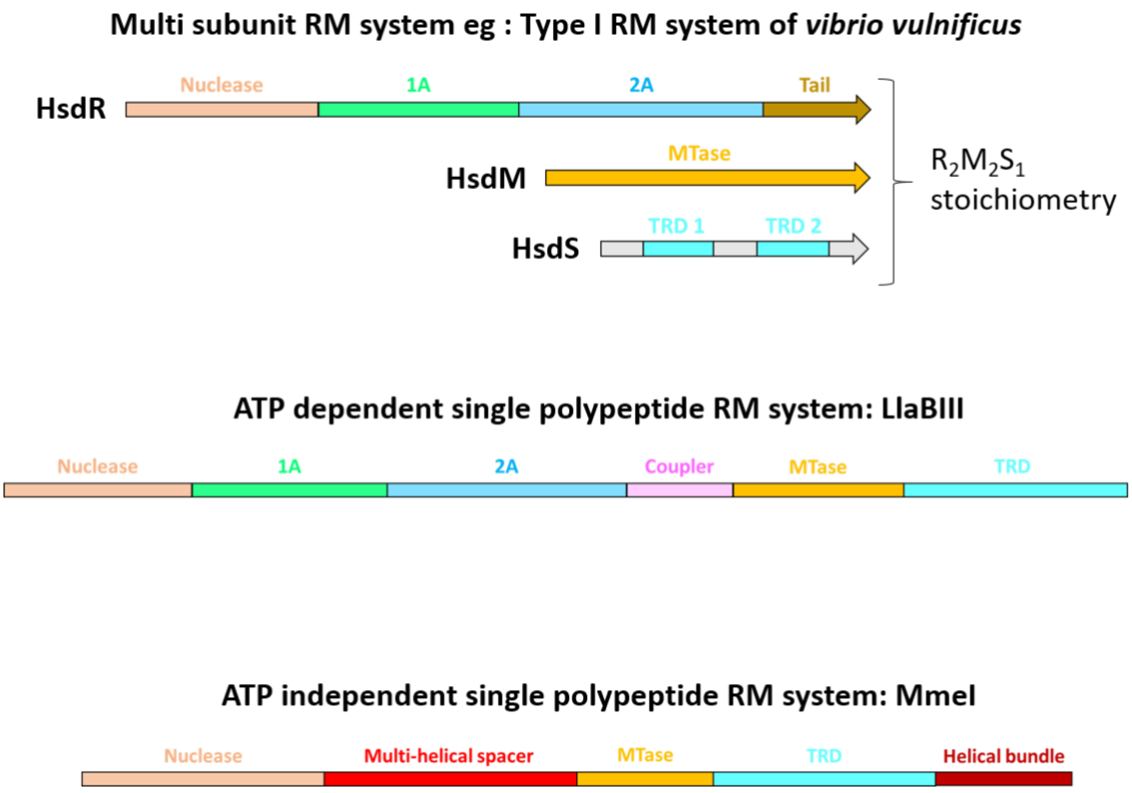

Supplementary figure S10. The primary domain architecture of a multi-subunit RM system (Type I RM system) and single polypeptide RM systems LlaBIII (Type ISP) and MmeI.

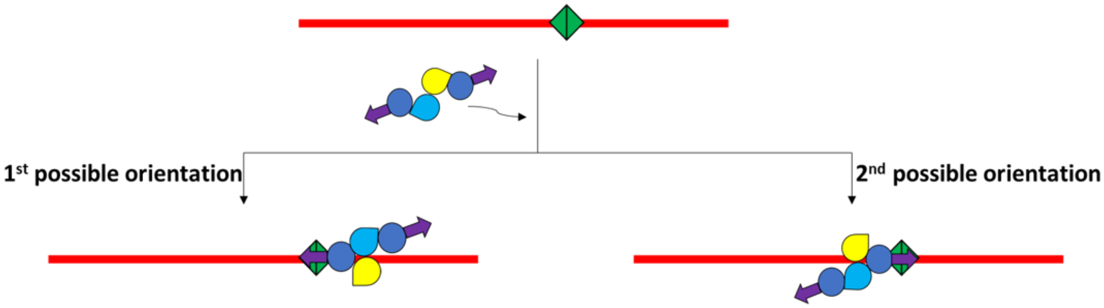

Supplementary figure S11. Possible orientations in which SauUSI can bind to a single-site substrate.

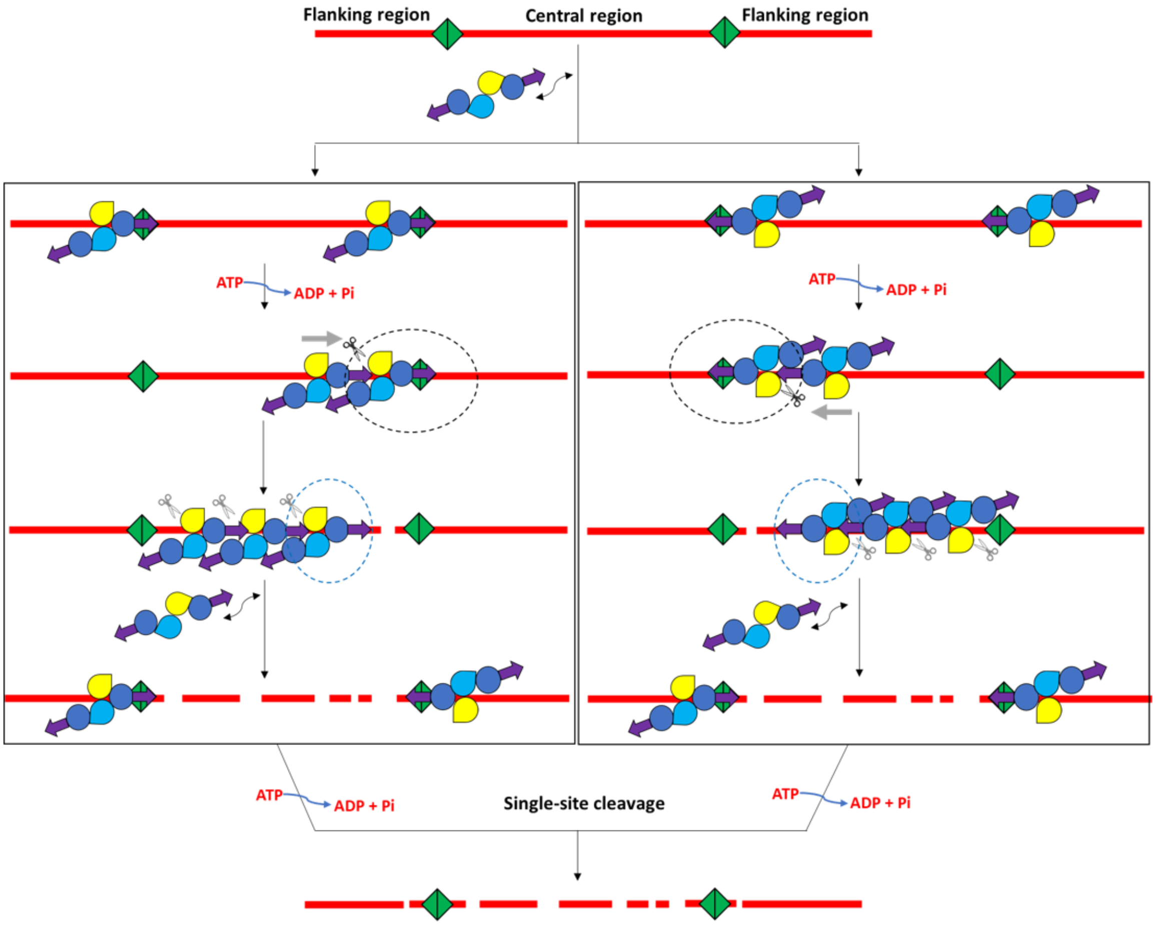

Supplementary figure S12. In the head-to-tail orientation, the first convergence can lead to stimulation of DNA cleavage (black pair of scissors) in the central region by the stationary SauUSI resulting in the enzyme falling off (black dotted circle). The stalled enzyme (blue dotted circle) acts as a roadblock for the subsequent pile-up. The inefficient cleavage (grey pairs of scissors) by the piled-up enzymes leads to random double strand breaks in between the two target sites. The fragments that are left with a single-site can be further cleaved by SauUSI rebinding to the target site.

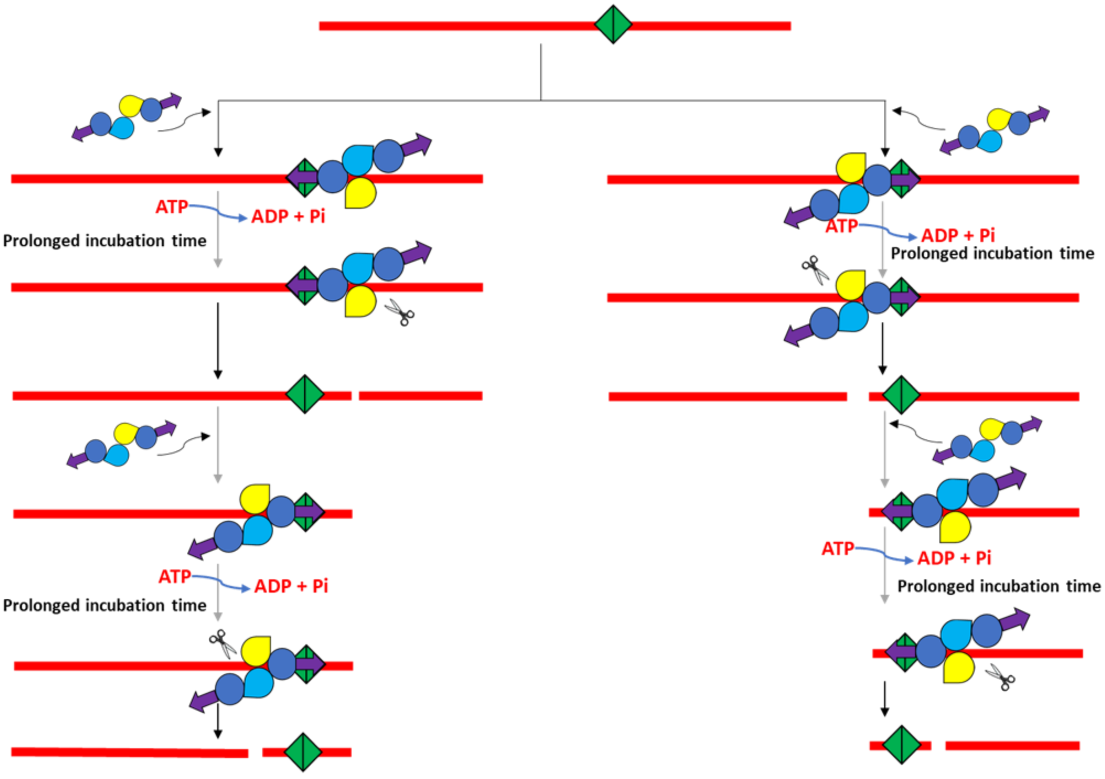

Supplementary figure S13. Irrespective of the orientation in which SauUSI binds to the target site in a single-site substrate, the resultant fragments are the same.

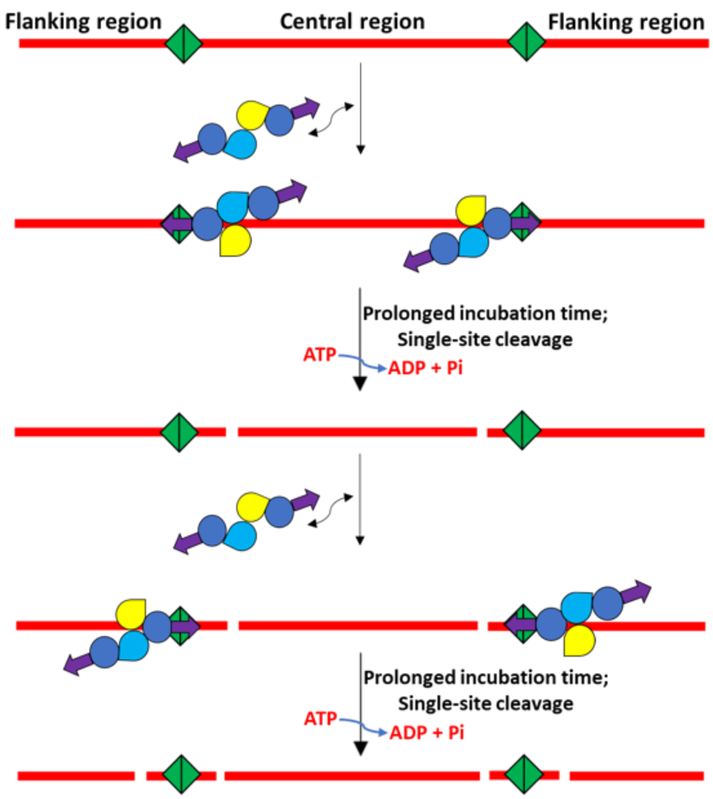

Supplementary figure S14. In the tail-to-tail orientation, convergence of enzymes is not possible. Cleavage in this case is similar to the cleavage of single-site substrate.

Supplementary table S1. Primers used in the study

Primer name	Sequence of primers (5'->3')
SauUSI_forward primer	GAAGGAGATATACATATGAGTAGATTACTAAATGATTTCAATC
SauUSI_reverse primer	GATGATGATGATGATGGGATCCATTTGTTAGATAACGATATATATC
SauUSI^{H119A} reverse primer	CTCAAAAATATATCCTTTGGCGGCGAATCCAGCAATAT
Triplex-site reverse primer	CACCTTCATCTCTGACATTTCTTTCTTCTTTCTTTGCTCGAGACCAGCTAAAATCCTGAAAGA
120 bp methylated forward primer	ATGAGACATGGTTTCTTGAGTGTATATGAACTATTCAAATCAGTGTCTTCGGACTACGC^{5m}CAGGAGAATTT
120 bp methylated reverse primer	ACGTGGTCAATAGCACAACAATGTCGTCACAAGCTCTTCACATCTATGCTAAATTCTC^{5m}CTGGCGTAGTCC
120 bp non-methylated forward primer	ATGAGACATGGTTTCTTGAGTGTATATGAACTATTCAAATCAGTGTCTTCGGACTACGCCAGGAGAATTT
120 bp non-methylated reverse primer	ACGTGGTCAATAGCACAACAATGTCGTCACAAGCTCTTCACATCTATGCTAAATTCTCCTGGCGTAGTCC
Three-site forward primer	GATGATACTTCCACTGGATCCAGGTTTGAAGTAACCAGAAAT
Sequencing forward primer	CACCACTTCAAGAACTCTGTAGCA
Sequencing reverse primer	CAAAATTATTTCTAGAGGGAAACCGT
EMSA methylated forward primer	GCCGCGGATCCTTCACAAGACGATTAC/5MedC/GGCGCTGAAAGACCAGCATATGCAATTTGACT
EMSA methylated reverse primer	AGTCAAATTGCATATGCTGGTCTTTCAGCG/5MedC/CGGTAATCGTCTTGTGAAGGATCCGCGGC
EMSA non-methylated forward primer	GCCGCGGATCCTTCACAAGACGATTACCGGCGCTGAAAGACCAGCATATGCAATTTGACT
EMSA non-methylated reverse primer	AGTCAAATTGCATATGCTGGTCTTTCAGCGCCGGTAATCGTCTTGTGAAGGATCCGCGGC

Supplementary table S2. Crystallographic data and refinement statistics.

Structure	SauUSI^{SELMET}
Space group	$P2_1$
Cell dimensions	
a, b, c (Å)	86.6 175.7 89.4
α, β, γ (deg)	90, 115.7, 90
Wavelength (Å)	0.9795
Resolution (Å) (Highest resolution shell)	87.8-3.1 (3.22-3.10)
R_{merge} (%) overall	0.08 (1.103)
I/σ	13.1 (1.6)
Completeness (%)	99.9 (100)
Redundancy	6.9 (7.1)
<u>Refinement</u>	
Resolution (Å)	56.8–3.1
No. of reflections	15469
R_{work}/R_{free} (%)	24.5/29.7
No. of atoms	12275
No. of ions	4
No. of water molecules	33
RMS deviations in	
Bond lengths (Å)	0.005
Bond angles (deg)	0.752
PDB ID	7CLG

Supplementary table S3. List of ORFs expressing SauUSI-like protein in different bacterial species. Sequences of these proteins were used to generate the phenogram shown in Supplementary figure 15.

**REase**	**Organism**	**Gram Status**	**Phylum**
SauUSI	*Staphylococcus aureus* subsp. *Aureus* USA300_FPR3757	Positive	Firmicutes
Ssi13838ORF2294P	*Staphylococcus simiae* NCTC13838	Positive	Firmicutes
Spa3CORF1910P	*Staphylococcus pasteuri* 3C	Positive	Firmicutes
Sep121ORFDP	*Staphylococcus epidermidis* CDC121	Positive	Firmicutes
SflMBTS1ORF7610P	*Staphylococcus fleurettii* MBTS-1	Positive	Firmicutes
SspOJ82ORF3790P	*Staphylococcus species* OJ82	Positive	Firmicutes
SmaS46ORF2218P	*Staphylococcus massiliensis* S46	Positive	Firmicutes
Slu700373ORF4825P	*Staphylococcus lutrae* ATCC 700373	Positive	Firmicutes
SchMU970ORF10704P	*Staphylococcus chromogenes* MU 970	Positive	Firmicutes
Sco11674ORFCP	*Staphylococcus condimenti*	Positive	Firmicutes
Ssi27848ORFCP	*Staphylococcus simulans*	Positive	Firmicutes
Sho575ORF6435P	*Staphylococcus hominis* FDAARGOS_575	Positive	Firmicutes
ShaK8ORFAP	*Staphylococcus haemolyticus* K8	Positive	Firmicutes
PciZF2ORF10855P	*Proteus cibarius* ZF2	Negative	Proteobacter
AspDF1ORF4285P	*Anthococcus species* DF1	Positive	Firmicutes
Avi4311ORF9560P	*Aerococcus viridans* CCUG4311	Positive	Firmicutes
Lra68ORF1585P	*Lactococcus raffinolactis* WiKim0068	Positive	Firmicutes
Cfupf3ORFBP	*Carnobacterium funditum* pf3	Positive	Firmicutes
Lla7266ORF1149P	*Lactococcus lactis* subsp. *Cremoris* P7266	Positive	Firmicutes
VspMN17ORF915P	*Vagococcus species* MN-17	Positive	Firmicutes
Vte21459ORF1820P	*Vagococcus teuberi*	Positive	Firmicutes
Eit15952ORFAP	*Enterococcus italicus* DSM 15952	Positive	Firmicutes
EfaSS17ORF2890P	*Enterococcus faecalis* S17	Positive	Firmicutes
EspPIfORF70P	*Enterococcus species* RIT-PI-f	Positive	Firmicutes
PpeFBL2ORFBP	*Pediococcus pentosaceus* FBL2	Positive	Firmicutes
LneY15ORF740P	*Lactobacillus nenjiangensis* SH-Y15	Positive	Firmicutes
Lsa74ORF4980P	*Lactobacillus sakei* WiKim0074	Positive	Firmicutes
Lal10ORF160110P	*Lactobacillus algidus* CMTALT10	Positive	Firmicutes
Pcl344ORFAP	*Pediococcus claussenii*	Positive	Firmicutes
PacBCC1ORF7875P	*Pediococcus acidilactici* BCC1	Positive	Firmicutes
PpeSS13ORF6945P	*Pediococcus pentosaceus*	Positive	Firmicutes
Khu618TORFAP	*Kurthia huakuii* LAM0618	Positive	Firmicutes
KmaJC30ORFAP	*Kurthia species* JC30 JC30T	Positive	Firmicutes
EspMN05ORFGP	*Enterococcus species* MN05	Positive	Firmicutes

Cdi1505ORF280036P	*Carnobacterium divergens* MFPA43A1505	Positive	Firmicutes
Ema16307ORFAP	*Exiguobacterium marinum*	Positive	Firmicutes
Esp31ORFAP	*Exiguobacterium* sp. GIC31	Positive	Firmicutes
HspKGW1ORFAP	*Halobacillus* species KGW1	Positive	Firmicutes
Gsp19038ORF3757P	*Geomicrobium* species	Positive	Firmicutes
PyaY32ORF14985P	*Pontibacillus yanchengensis* Y32	Positive	Firmicutes
Psp737ORF3620P	*Paenibacillus* species FSL H7-0737	Positive	Firmicutes
Cma28ORF2962P	*Carnobacterium maltaromaticum* LMA28	Positive	Firmicutes
ShaB36ORF2460P	*Salinicoccus halodurans* H3B36	Positive	Firmicutes
VluLBD1ORF1493P	*Vagococcus lutrae* LBD1	Positive	Firmicutes
Ksi49154ORF4925P	*Kurthia sibirica* ATCC 49154	Positive	Firmicutes
Kzo404ORF2926P	*Kurthia zopfii* NCTC404	Positive	Firmicutes
Bth3070ORF20169P	*Brochothrix thermosphacta* isolate EBP 3070	Positive	Firmicutes
Ssi12223ORF735P	*Solibacillus silvestris*	Positive	Firmicutes
Bsp313ORFBP	*Bacillus* species m3-13	Positive	Firmicutes
Bsp22090ORF14555P	*Bacillus* species FJAT-22090	Positive	Firmicutes
Psp10380ORF25130P	*Paenibacillus* species IHBB 10380	Positive	Firmicutes
Ppi16418ORF2896P	*Paenibacillus pini*	Positive	Firmicutes
BpsOF4ORF17150P	*Bacillus pseudofirmus* OF4	Positive	Firmicutes
Psp14ORFBP	*Paenisporosarcina* species TG-14	Positive	Firmicutes
Pko701ORF1785P	*Planococcus kocurii* HK 701	Positive	Firmicutes
PriM8ORF12315P	*Planococcus rifietoensis* M8	Positive	Firmicutes
Bco26ORF2542P	*Bacillus coagulans* 2-6	Positive	Firmicutes
SspHYO08ORF8335P	*Sporosarcina* species HYO08	Positive	Firmicutes
SurP8ORF8955P	*Sporosarcina ureae* P8	Positive	Firmicutes
Ssp2304ORF13325P	*Sporosarcina* species PTS2304	Positive	Firmicutes
SaiMSP4ORFBP	*Salinibacillus aidingensis* MSP4	Positive	Firmicutes
BmaEB01ORFAP	*Bacillus* species EB01	Positive	Firmicutes
Osp160ORF19460P	*Oceanobacillus* species 160	Positive	Firmicutes
Vpa21DORF1561P	*Virgibacillus pantothenticus* 21D	Positive	Firmicutes
OpiS1ORF1431P	*Oceanobacillus picturae* S1	Positive	Firmicutes
BmeNi23ORF30785P	*Bacillus megaterium* Ni2-3	Positive	Firmicutes
Bsp25496ORF12975P	*Bacillus* species FJAT-25496	Positive	Firmicutes
BcyCH4ORF10485P	*Bacillus cytotoxicus* CH_4	Positive	Firmicutes
Bce938ORF869P	*Bacillus cereus* RIVM_BC938	Positive	Firmicutes
Dpi3069ORF8625P	*Dolosigranulum pigrum* KPL3069	Positive	Firmicutes
SsaK12ORF1459P	*Streptococcus salivarius* K12	Positive	Firmicutes
Ssp431ORF1390P	*Streptococcus* species oral taxon 431	Positive	Firmicutes
Sau641ORFAP	*Streptococcus australis* ATCC 700641	Positive	Firmicutes
Sin779ORFAP	*Streptococcus infantis* ATCC 700779	Positive	Firmicutes
Ama81211ORFAP	*Alloscardovia macacae* UMA81211	Positive	Actinobacteria

Aom7705AORF381P	*Alloscardovia omnicolens* CMW7705A	Positive	Actinobacteria
Ssu66ORF451P	*Streptococcus suis* LSS66	Positive	Firmicutes
Sph51973ORF2830P	*Streptococcus phocae*	Positive	Firmicutes
SubORFAP	*Streptococcus uberis* 0140J	Positive	Firmicutes
SinSi1ORF3425P	*Streptococcus iniae* UEL-Si1	Positive	Firmicutes
Spo176ORFAP	*Streptococcus porcinus* Jelinkova 176	Positive	Firmicutes
Bau1417ORF1575P	*Brevibacterium aurantiacum* SMQ-1417	Positive	Actinobacteria
Cgl56828ORF6225P	*Corynebacterium glutamicum* USDA-ARS-USMARC-56828	Positive	Actinobacteria
Cha44683ORF5845P	*Corynebacterium halotolerans* YIM 70093	Positive	Actinobacteria
WspKH42ORF8925P	*Weissella* species 26KH-42	Positive	Firmicutes
Lfa3681ORF1420P	*Lactobacillus farciminis*	Positive	Firmicutes
Wci103ORF3080P	*Weissella cibaria* DmW_103	Positive	Firmicutes
LgaPS3ORF22077P	*Lactobacillus gastricus* PS3	Positive	Firmicutes
Lru27782ORF5640P	*Lactobacillus ruminis* ATCC 27782	Positive	Firmicutes
Leq6820ORF1664P	*Lactobacillus equi* DPC 6820	Positive	Firmicutes
LsaGJ24ORFAP	*Lactobacillus salivarius* GJ-24	Positive	Firmicutes
LagLa3ORF8730P	*Lactobacillus agilis* La3	Positive	Firmicutes
Lin209ORFAP	*Lactobacillus ingluviei* Autruche 4	Positive	Firmicutes
LmuMORFCP	*Lactobacillus mucosae* LM1	Positive	Firmicutes
Lre53608ORF581P	*Lactobacillus reuteri* ATCC 53608	Positive	Firmicutes
LmuEF1ORFFP	*Lactobacillus murinus* EF-1	Positive	Firmicutes
LacP2ORFAP	*Lactobacillus acidophilus* P2	Positive	Firmicutes
Ljo10ORF5995P	*Lactobacillus johnsonii* ZLJ010	Positive	Firmicutes
Lgi2485ORF6560P	*Lactobacillus gigeriorum* CRBIP 24.85	Positive	Firmicutes
Lac1533ORF561P	*Lactobacillus acidipiscis* ACA-DC 1533	Positive	Firmicutes
Lpo301ORFDP	*Lactobacillus pobuzihii* E100301	Positive	Firmicutes
Lma3596ORFDP	*Lactobacillus mali* KCTC 3596	Positive	Firmicutes
Lvi20605ORFCP	*Lactobacillus vini*	Positive	Firmicutes
Lna11827ORF10790P	*Lactobacillus nagelii* TMW 1.1827	Positive	Firmicutes
Lsa30AORF7709P	*Lactobacillus saerimneri* 30a	Positive	Firmicutes
LsaSORF1453P	*Lactobacillus salivarius* UCC118	Positive	Firmicutes
Lje7AUSORFAP	*Lactobacillus jensenii* SJ-7A-US	Positive	Firmicutes
Lho179TORF5760P	*Lactobacillus hominis* CRBIP 24.179	Positive	Firmicutes
Ljo33200ORFAP	*Lactobacillus johnsonii* ATCC 33200	Positive	Firmicutes
LorJ1ORF300P	*Lactobacillus oris* J-1	Positive	Firmicutes
Lre100ORF5017P	*Lactobacillus reuteri* 100-23	Positive	Firmicutes
Lgi2485ORF5145P	*Lactobacillus gigeriorum* CRBIP 24.85	Positive	Firmicutes
Lam11664ORFAP	*Lactobacillus amylolyticus* DSM 11664	Positive	Firmicutes
Lap185ORF335P	*Lactobacillus apis* ESL0185	Positive	Firmicutes
LacFSI4ORF57P	*Lactobacillus acidophilus* FSI4	Positive	Firmicutes

Lcr125ORF1057P	*Lactobacillus crispatus* 125-2-CHN	Positive	Firmicutes
Lul16047ORFAP	*Lactobacillus ultunensis* DSM 16047	Positive	Firmicutes
LkeZW3ORF1630P	*Lactobacillus kefiranofaciens* ZW3	Positive	Firmicutes
Erh8163ORF595P	*Erysipelothrix rhusiopathiae* NCTC8163	Positive	Firmicutes
Dfa26099ORFAP	*Dielma fastidiosa* strain JC13	Positive	Firmicutes
Msm2374ORF2340P	*Methanobrevibacter smithii* DSM 2374	Archaea	Euryachaeota
MspJH1ORFAP	*Methanobrevibacter* species JH1	Archaea	Euryachaeota
Eba102ORF620P	*Erysipelotrichaceae* bacterium SG0102	Positive	Firmicutes
Lsp6250ORFAP	*Longibaculum* species KGMB06250	Positive	Firmicutes
CspD7ORFAP	*Coprobacillus* species D7	Positive	Firmicutes
Cba70BAORF2459P	*Clostridiales bacterium* 70B-A	Positive	Firmicutes
MhoHB1ORF1217P	*Methanosarcina horonobensis* HB-1	Archaea	Euryachaeota
Cvi10228ORF20690P	*Clostridium vincentii* DSM 10228	Positive	Firmicutes
Hhi503ORF897P	*Hathewaya histolytica* NCTC503	Positive	Firmicutes
CboE1ORFAP	*Clostridium botulinum* E1 BoNT E Beluga	Positive	Firmicutes
CalB3ORFAP	*Clostridium algidicarnis* strain B3	Positive	Firmicutes
MelInORF4925P	*Megasphaera elsdenii indica*	Negative	Firmicutes
Psp12067ORF680P	*Phascolarctobacterium* species YIT 12067	Negative	Firmicutes
Pma2259ORFAP	*Prochlorococcus marinus* clone ASNC2259	Negative	Cyanobacteria
Asp04ORF7325P	*Acinetobacter* species TTH0-4	Negative	Proteobacter
Cle5427ORF238P	*Clostridium lentocellum*	Negative	Firmicutes
Pba547ORF5246P	*Planctomycetes bacterium* SRT547	Negative	Planctomycetes
Pin37ORF2683P	*Psychromonas ingrahamii* 37	Negative	Proteobacter
TmaKORJJORF9230P	*Tenacibaculum maritimum* TM-KORJJ	Negative	Bacteroidetes
MspA21ORF507P	*Myroides* species A21	Negative	Bacteroidetes
AleSS8ORF3884P	*Algibacter lectus*	Negative	Bacteroidetes
Cal14237ORF2759P	*Cellulophaga algicola* DSM 14237	Negative	Bacteroidetes
Ksp4hORF2830P	*Krokinobacter* species 4H-3-7-5	Negative	Bacteroidetes
TdiTDORF12160P	*Tenacibaculum dicentrarchi* AY7486TD	Negative	Bacteroidetes
Wfu1127ORF6300P	*Wenyingzhuangia fucanilytica* CZ1127	Negative	Bacteroidetes
BspC07ORF6720P	*Balneola* species EhC07	Negative	Bacteroidetes
DtiY6201ORFBP	*Dyadobacter tibetensis* Y620-1	Negative	Bacteroidetes
Nsp7ORF12030P	*Nonlabens* species MB-3u-79	Negative	Bacteroidetes
GspJM1ORFFP	*Gillisia* sp. JM1	Negative	Bacteroidetes
FfaWV33ORF12875P	*Flavobacterium faecale* WV33	Negative	Bacteroidetes
Ssa1343ORF9240P	*Spiroplasma sabaudiense* Ar-1343	Cell wall less	Tenericutes
Stu4cORF740P	*Spiroplasma turonicum* Tab4c	Cell wall less	Tenericutes
Sdi1ORF5590P	*Spiroplasma diminutum* CUAS-1	Cell wall less	Tenericutes
DauHRMORFAP	*Desulfobacterium autotrophicum* HRM2	Negative	Proteobacter
AcaWCW12ORF12300P	*Aeromonas caviae* WCW1-2	Negative	Proteobacter
ZdeF131ORF5985P	*Zobellella denitrificans* F13-1	Negative	Proteobacter

Opr1222ORF12495P	*Oceanisphaera profunda* SM1222	Negative	Proteobacter
Van11008ORF4200P	*Vibrio anguillarum* CNEVA NB11008	Negative	Proteobacter
Plu542ORF22150P	*Pseudoalteromonas luteoviolacea* S40542	Negative	Proteobacter
Vch223ORF13130P	*Vibrio cholerae* FDAARGOS_223	Negative	Proteobacter
VcoL11ORFCP	*Vibrio cholerae* L11	Negative	Proteobacter
Pin37ORF2523P	*Psychromonas ingrahamii* 37	Negative	Proteobacter
PspND6BORF48P	*Pseudoalteromonas* species ND6B	Negative	Proteobacter
PspP19ORF2558P	*Pseudoalteromonas* species P1-9	Negative	Proteobacter
Gpu611ORF1206P	*Glaciecola punicea* DSM 14233	Negative	Proteobacter
Msp5810ORF16510P	*Marinobacterium* species ST58-10	Negative	Proteobacter
AspLTRORFBP	*Alteromonas* species LTR	Negative	Proteobacter
AmeRG65ORF575P	*Alteromonas mediterranea* RG65	Negative	Proteobacter
AnaSN2ORF18570P	*Alteromonas naphthalenivorans* SN2	Negative	Proteobacter
Psh73ORF3029P	*Plesiomonas shigelloides* 302-73	Negative	Proteobacter
IspA28LORFAP	*Idiomarina* species A28L	Negative	Proteobacter
PdaNa1ORF2280P	*Photobacterium damselae* subsp. *Damselae* KC-Na-1	Negative	Proteobacter
UpbMLORFCP	Unknown delta proteobacterium MLMS-1	Negative	Proteobacter
Mal20ZORF1181P	*Methylomicrobium alcaliphilum* 20Z	Negative	Proteobacter
Tni14787ORF3285P	*Thioalkalivibrio nitratireducens*	Negative	Proteobacter
TspS2ORF17329P	*Thauera aminoaromatica* S2	Negative	Proteobacter
JspCG3ORFGP	*Janthinobacterium* species CG3	Negative	Proteobacter
HhaAORF2025P	*Halorhodospira halochloris* A	Negative	Proteobacter
MspES1ORF9400P	*Marinobacter* species ES-1	Negative	Proteobacter
Mal893ORFAP	*Marinobacter algicola* DG893	Negative	Proteobacter
Msp20148ORF1085P	*Marinobacter* species BSs20148	Negative	Proteobacter
SspGrORF853P	*Spirochaeta* species Grapes	Negative	Spirochetes
AUR51707.1 NgoFVII family restriction endonuclease [Neisseriaceae bacterium DSM 100970]	*Neisseria*	Negative	Proteobacter
Fmo9817SORF785P	*Fusobacterium mortiferum* ATCC 9817S	Negative	Fusobacteria
Ful12112ORF246P	*Fusobacterium ulcerans* NCTC12112	Negative	Fusobacteria
Fpe1283ORF6075P	*Fusobacterium periodonticum* KCOM 1283	Negative	Fusobacteria
